# The prognostic value of preoperative peripheral blood inflammatory biomarkers in extrahepatic cholangiocarcinoma: a systematic review and meta-analysis

**DOI:** 10.3389/fonc.2024.1437978

**Published:** 2024-08-29

**Authors:** Di Zeng, Yaoqun Wang, Ningyuan Wen, Jiong Lu, Bei Li, Nansheng Cheng

**Affiliations:** ^1^ Division of Biliary Surgery, Department of General Surgery, West China Hospital, Sichuan University, Chengdu, Sichuan, China; ^2^ Research Center for Biliary Diseases, West China Hospital, Sichuan University, Chengdu, Sichuan, China

**Keywords:** neutrophil-to-lymphocyte ratio, platelet-to-lymphocyte ratio, lymphocyte to monocyte ratio, extrahepatic cholangiocarcinoma, meta-analysis

## Abstract

**Background:**

Recent evidence indicates that inflammation plays a major role in the pathogenesis and progression of CCA. This meta-analysis seeks to evaluate the prognostic implications of preoperative inflammatory markers, specifically NLR, PLR, and LMR, in patients with eCCA. By focusing on these preoperative biomarkers, this study aims to provide valuable insights into their prognostic value and potential utility in clinical practice.

**Methods:**

For this analysis, comprehensive searches were conducted in PubMed, Embase, and Web of Science databases from inception to May 2024. The primary outcomes of interest focused on the association between the levels of NLR, PLR, and LMR and the prognosis of eCCA patients. Statistical analyses were conducted using STATA 17.0 software.

**Results:**

The meta-analysis, involving 20 retrospective studies with 5553 participants, revealed significant correlations between preoperative biomarkers and the prognosis of eCCA patients. Elevated NLR, PLR, and decreased LMR levels were extensively studied regarding overall survival (OS) in eCCA patients. Elevated NLR was an independent predictor of poor OS (HR 1.86, p < 0.001), similar to elevated PLR (HR 1.76, p < 0.001), while decreased LMR predicted poor OS (HR 2.16, p < 0.001). Subgroup analyses based on eCCA subtypes and curative surgery status showed consistent results.

**Conclusions:**

In conclusion, our study emphasizes the clinical significance of assessing NLR, PLR, and LMR preoperatively to predict patient prognosis. Elevated NLR and PLR values, along with decreased LMR values, were linked to poorer overall survival (OS). Large-scale prospective cohort studies are required to confirm their independent prognostic value in eCCA.

**Systematic review registration:**

https://www.crd.york.ac.uk/prospero/, identifier CRD42024551031.

## 1 Introduction

Cholangiocarcinoma (CCA) is an exceptionally aggressive cancer arising from the biliary duct epithelium (1) and is the second most common primary liver tumor, accounting for 5 to 30% of all primary liver malignancies (2). Cholangiocarcinoma can be classified based on its anatomical location into intrahepatic CCA (iCCA) and extrahepatic CCA (eCCA), each with distinct pathophysiological characteristics and clinical outcomes (3). Extrahepatic cholangiocarcinoma, which includes perihilar and distal cholangiocarcinoma, is one of the most unfavorable cancer diagnoses due to its aggressive nature (4). Despite advances in surgical techniques, perioperative management, and postoperative treatments, oncological outcomes remain poor after curative resection, likely due to the lack of effective additional therapies and predictive biomarkers for treatment response (5).

Etiological and experimental evidence indicates that inflammation plays a major role in the pathogenesis and progression of CCA (6). Increasing evidence suggests that cancer-associated inflammation is involved in numerous cancer-related processes, including initiation, progression, and metastasis (7). The condition of cancer patients can be reflected by complete blood count (CBC) markers, such as neutrophils, platelets, and lymphocytes (8). The neutrophil-lymphocyte ratio (NLR), defined as the ratio of absolute neutrophil count to absolute lymphocyte count, has been found to be closely related to survival and recurrence in hepatocellular carcinoma (HCC). Numerous studies have confirmed that inflammatory markers, such as the neutrophil-to-lymphocyte ratio (NLR) and platelet-to-lymphocyte ratio (PLR), are associated with the prognosis of various cancers, including hepatocellular carcinoma (HCC) (9), gallbladder carcinoma (10), and pancreatic cancer (11). Similarly reported, the Lymphocyte to Monocyte Ratio (LMR) has shown considerable promise as a prognostic indicator across a spectrum of tumor types, encompassing lymphoma (12), colorectal cancer (13), and lung cancer (14). Nevertheless, a comprehensive summary of the prognostic significance of preoperative biomarkers, including NLR, PLR, and LMR, in extrahepatic cholangiocarcinoma is currently lacking. Furthermore, the correlation between peripheral blood inflammatory indicators and the prognosis of CCA (cholangiocarcinoma) remains to be further explored.

In this study, our focus lay on assessing the prognostic significance of preoperative NLR, PLR, and LMR specifically in eCCA (extrahepatic cholangiocarcinoma). Previous meta-analyses have examined the role of inflammatory factors in predicting prognosis among cholangiocarcinoma patients. However, these analyses either concentrated on the entire cholangiocarcinoma cohort (15), or specifically targeted iCCA (16). This is the first meta-analysis to investigate the prognostic value of preoperative NLR, PLR, and LMR in eCCA, offering valuable insights for prognostic prediction in these patients.

## 2 Materials and methods

### 2.1 Literature search

This systematic review was registered in the International Prospective Register of Systematic Reviews (PROSPERO) with the ID CRD42024534979 and conducted in accordance with the PRISMA (Preferred Reporting Items for Systematic Reviews and Meta-Analyses) criteria. Two independent reviewers, Zeng D and Wen NY, conducted searches on PubMed, Embase, and Web of Science from their inception until April 2024, restricting the search results to English. In cases of disagreement between Zeng D and Wen NY, a third reviewer, Wang YQ, was consulted to reach a consensus.

### 2.2 Inclusion and exclusion criteria

Inclusion criteria:

1. Patients diagnosed with cholangiocarcinoma (CCA) through pathological examination. 2. Studies were eligible for inclusion if they examined how preoperative biomarkers like NLR, PLR, and LMR correlate with patient outcomes in terms of prognosis. 3. The studies furnished hazard ratios (HR) alongside their respective 95% confidence intervals (CI), elucidating the influence of preoperative biomarkers like NLR, PLR, and LMR on patient overall survival (OS). 4. Studies in which biomarkers were measured before any chemotherapy or radiotherapy, to ensure baseline values were not influenced by these interventions.

Exclusion criteria:

1. Patients pathologically diagnosed with benign or borderline cholangiocarcinoma (CCA) or other gastrointestinal tumors were excluded. 2. Studies that did not assess at least one of the following preoperative biomarkers—NLR, PLR, or LMR—were excluded. 3. Studies lacking sufficient data, including those without reported postoperative survival times or essential statistics such as hazard ratios (HR), odds ratios (OR), or relative risks (RR), were excluded. 4. Studies that did not focus exclusively on extrahepatic cholangiocarcinoma (eCCA), or that combined eCCA with other cancer types, which may confound the specific prognostic value of the biomarkers for eCCA.

### 2.3 Statistical analysis

Survival data were analyzed using hazard ratios (HRs) and their corresponding 95% confidence intervals (CIs) through multivariate regression analysis, while categorical variables were assessed using odds ratios (ORs). Statistical heterogeneity was evaluated using Cochrane’s Q-test and I² statistics, with predefined thresholds for low, moderate, and high heterogeneity set at 25%, 50%, and 75%, respectively. A random-effects model was employed regardless of the heterogeneity level.

Subsequent subgroup analyses were performed to explore potential sources of heterogeneity, including the location of extrahepatic cholangiocarcinoma (hilar and distal), stratified by the cutoff values of NLR, PLR, and LMR. A significance level of P < 0.05 was considered statistically significant. Publication bias was assessed using funnel plots and Egger’s test. All statistical analyses were conducted using STATA 17.0 software.

### 2.4 Quality assessment of studies

Two independent investigators, Zeng D and Wen NY, evaluated the quality of the included studies using the Newcastle–Ottawa Scale (NOS), which assesses aspects such as case selection, cohort comparison, and exposure risk assessment. Only studies scoring six or higher on the NOS were included in the final meta-analysis.

## 3 Results

### 3.1 Literature search

**Figure 1:**
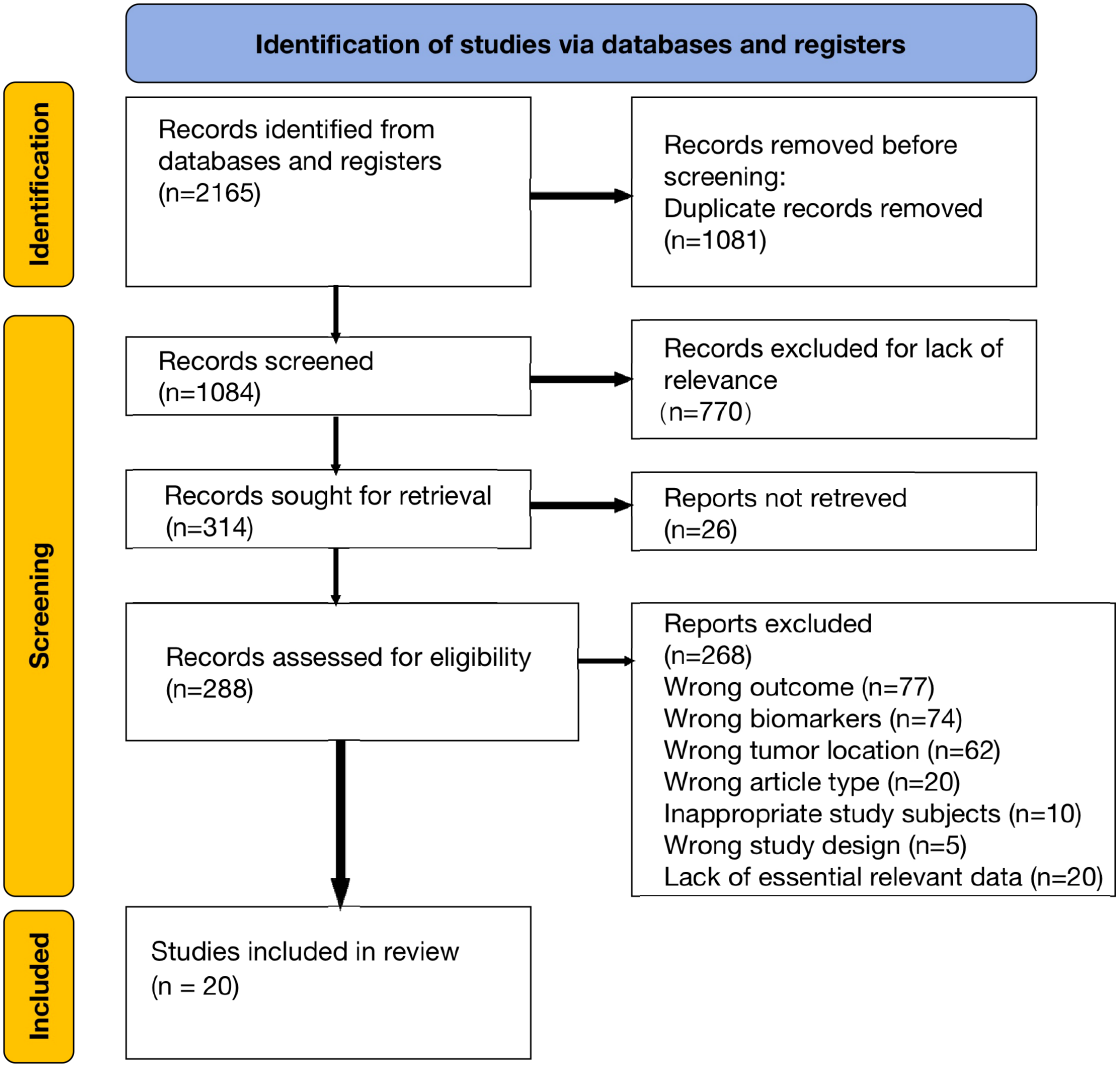
PRISMA flowchart for literature collection and screening inclusion.

We initially identified 2165 articles from electronic databases (PubMed, Embase, and Web of Science). After removing duplicates and irrelevant studies, we assessed 288 full-text articles for eligibility. Following a thorough examination, 20 studies were deemed eligible for qualitative synthesis. The basic characteristics of these included studies are presented in **Table 1**. The article selection process is illustrated in the PRISMA diagram below (**Figure 1**).

**Table 1:** Details of included original studies in this meta-analysis.

Author	Year	Country	patients number	tumor	NLR				PLR				LMR			
					HR	LL	UL	cutoff	HR	LL	UL	cutoff	HR	LL	UL	cutoff
Hoshimoto 2019	2019	Japan	53	distal CCA	1.487	0.67	3.301	1.966	2.084	0.989	4.389	187.8	1.691	0.76	3.764	4.633
Kota Sahara 2021	2021	Japan	245	distal CCA	2.84	1.57	4.88	9	1.28	0.75	2.1	266				
Fengming Ji 2020	2020	China	59	distal CCA	2.81	1.099	7.1847	2.933	2.18	0.9032	5.2616	270				
Kumamoto 2018	2018	Japan	84	distal CCA	6.77	2.44	21.13	2								
Terasaki 2021	2021	Japan	140	distal CCA	1.55	1	2.4	4.5	4.01	1.7534	9.1707	300				
Miyahara 2020	2020	Japan	40	distal CCA	11.81	3.296	42.32	3.14					4.837	1.826	12.81	4.55
So Jeong Yoon 2022	2022	Korean	1219	distal CCA	0.992	0.893	1.102	N/A								
Yuki Kitano 2019	2019	Japan	110	eCCA	1.44	0.77	2.57	2.93	1.71	0.97	2.89	185				
Yuki Kitano 2017	2017	Korean	120	eCCA	1.32	0.8	2.13	2.8	1.85	1.13	2.95	185				
W. Beal 2016	2016	USA	1092	eCCA	2.08	1.35	3.21	5								
Pieter Saragih 2022	2022	Indonesia	115	eCCA	1.844	1.19	2.848	5.5								
Matsumoto 2024	2024	Japan	182	eCCA	1.04	0.62	1.76	3	1.21	0.75	1.93	150				
Shijie Li 2022	2022	China	140	eCCA	2.12	1.4	3.23	2.66	1.75	1.1	2.78	125.82	1.54	1.02	2.31	2.86
Okumura 2015	2015	Japan	207	eCCA	1.473	0.743	2.635	4								
Toyoda 2022	2022	USA;Japan	485	eCCA	1.39	1.12	1.74	4	1.07	0.84	1.36	150				
Wang 2021	2021	China	94	hilar CCA	2.78	1.59	4.86	3.6	2.27	1.22	4.24	N/A				
Zhiqiang Lin 2022	2022	China	76	hilar CCA	1.147	0.455	4.404	2					5.525	1.5048	20.2851	4.02
MingYang Ge 2023	2023	China	333	hilar CCA	1.941	1.491	2.525	1.68	2.883	2.196	3.783	113.1				
Nechita 2022 I	2022	Romania	72	hilar CCA	1.8	0.97	3.32	3	1.82	0.94	3.55	150	1.887	1.0872	3.2752	3
Nechita 2022 II	2022	Romania	72	hilar CCA	3.45	1.81	6.58	6	1.238	0.68	2.23	200				

### 3.2 Study characteristics and quality assessment

The meta-analysis included a total of 5,553 patients diagnosed with extrahepatic cholangiocarcinoma, with studies spanning publication years from 2015 to 2024. Concerning preoperative biomarkers, 20 studies investigated the impact of NLR on prognosis, 15 studies examined the impact of PLR, and 5 studies explored the impact of LMR on prognosis. The Newcastle–Ottawa scale ranged from X to X, signifying an overall high quality of methodology in the included studies (**Table 2**).

**Table 2:** Newcastle-Ottawa Scale (NOS) scoring criteria for original studies included in this meta-analysis.

study	selection				comparability	Outcome			NOS score
	Representativeness of the exposed cohort	Selection of the non-exposed cohort	Ascertainment of exposure	Demonstration that outcome of interest was not present at start of study	Comparability of cohorts based on the design or analysis	Assessment of outcome	Was follow-up long enough for outcomes to occur	Adequacy of follow up of cohorts	
Hoshimoto 2019	★	★	★	★	★	★	★	★	8
Kota Sahara 2021	★	★	★	★	★	★	★	★	8
Fengming Ji 2020	★	★	★	★	★	★	★	–	7
Kumamoto 2018	★	★	★	★	★	★	★	–	7
Terasaki 2021	★	★	★	★	★	★	★	★	8
Miyahara 2020	★	★	★	★	★	★	★	★	8
So Jeong Yoon 2022	★	★	★	★	★	★	★	★	8
Yuki Kitano 2019	★	★	★	★	★	–	★	★	7
Yuki Kitano 2017	★	★	★	★	★	★	★	★	8
W. Beal 2016	★	★	★	★	★	★	★	★	8
Pieter Saragih 2022	★	★	★	★	★	–	★	★	7
Matsumoto 2024	★	★	★	★	★	★	★	★	8
Shijie Li 2022	★	★	★	★	★	★	★	★	8
Okumura 2015	★	★	★	★	★	–	★	★	7
Toyoda 2022	★	★	★	★	★	–	★	★	7
Wang 2021	★	★	★	★	★	★	★	★	8
Zhiqiang Lin 2022	★	★	★	★	★	★	★	★	8
MingYang Ge 2023	★	★	★	★	★	★	–	★	7
Nechita 2022 I	★	★	★	★	★	★	★	★	8
Nechita 2022 II	★	★	★	★	★	★	★	★	8

Symbol ★ indicates that this item has been included in the analysis within this study.

### 3.3 Correlation between the NLR and OS of eCCA patients

**Figure 2:**
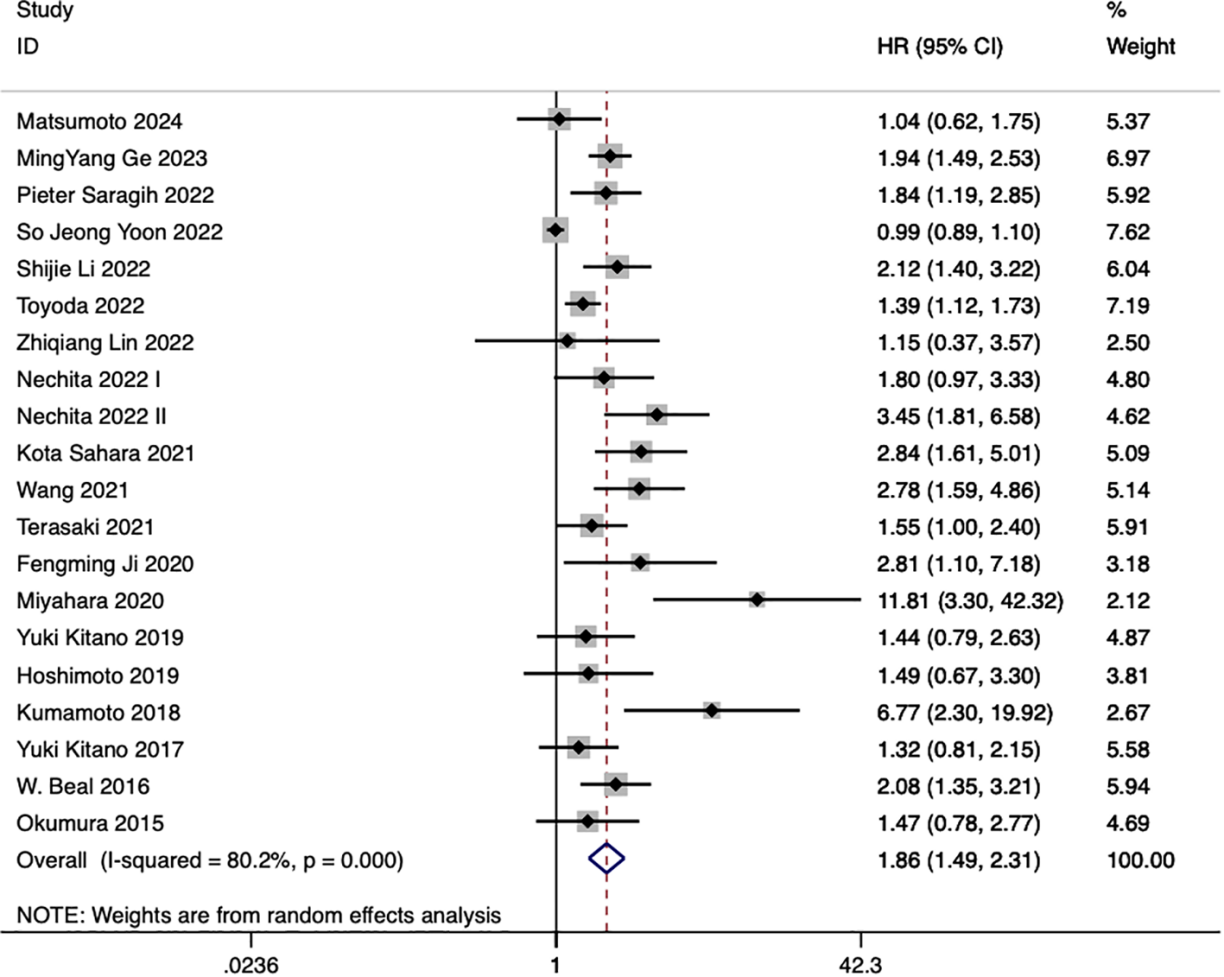
Impact of NLR on overall survival in eCCA patients,.

20 studies discussed the relationship between NLR and eCCA prognosis. In 12 studies, an elevated NLR was found to be an independent predictor of impaired OS among patients with eCCA, while in 8 studies, the influence of elevated NLR on OS did not attain statistical significance. The combined analysis of all 20 publications showed that NLR values higher than the defined cut-off values predicted a worse OS (HR 1.86, 95% CI 1.49–2.31, p < 0.001) with high heterogeneity (**Figure 2**).

### 3.4 Correlation between the PLR and OS of eCCA patients

**Figure 3:**
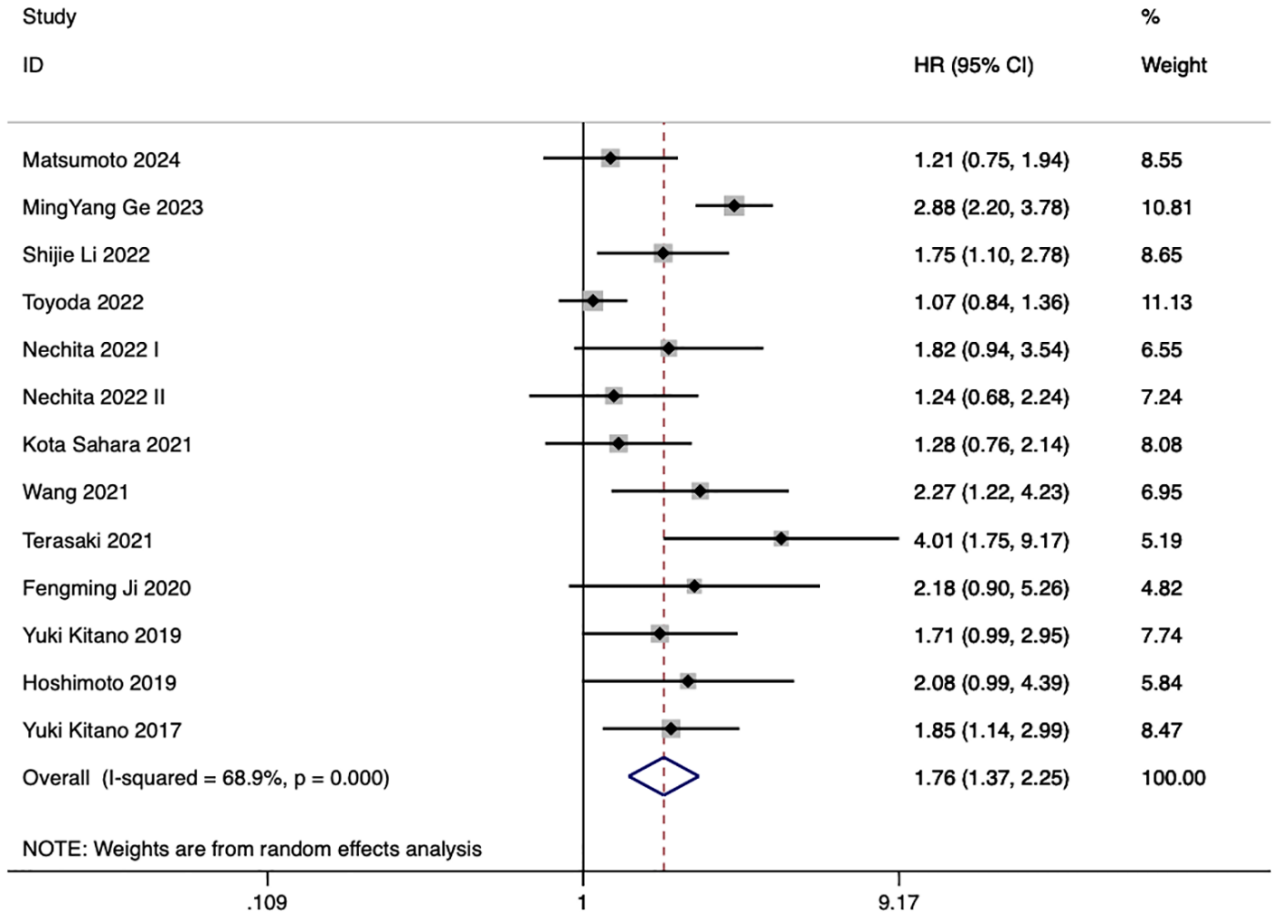
Impact of PLR on overall survival in eCCA patients.

13 studies examined the correlation between PLR and eCCA prognosis. In five studies, an elevated PLR emerged as an independent predictor of compromised OS among patients with eCCA. Conversely, in 8 studies, the impact of elevated PLR on OS did not achieve statistical significance. The pooled analysis of all twenty publications revealed that PLR values exceeding the defined cut-off values were indicative of poorer OS (HR 1.76, 95% CI 1.37–2.25, p < 0.001) despite encountering high heterogeneity (**Figure 3**).

### 3.5 Correlation between the LMR and OS of eCCA patients

**Figure 4:**
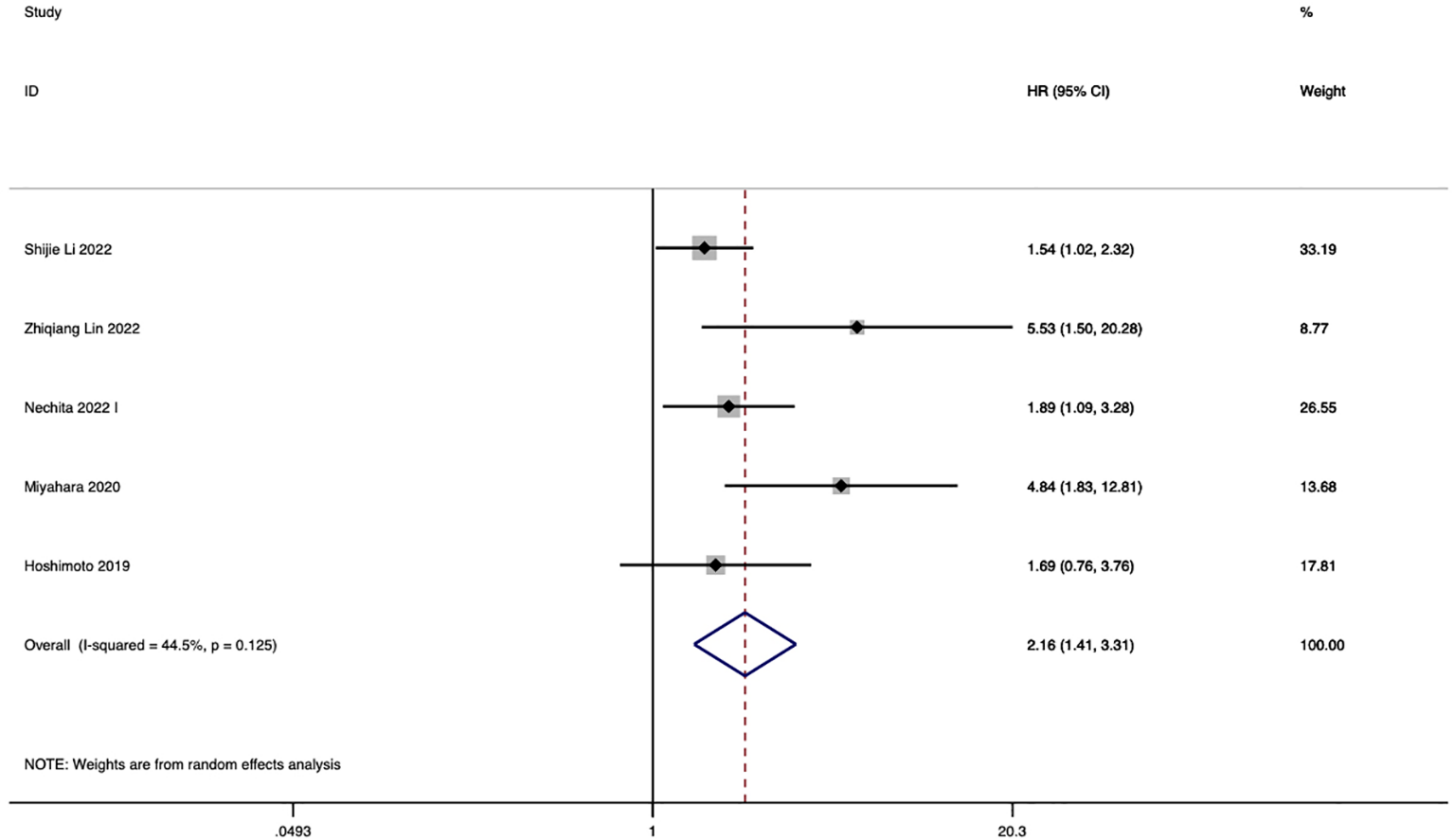
Impact of LMR on overall survival in eCCA patients.

**Figure 5:**
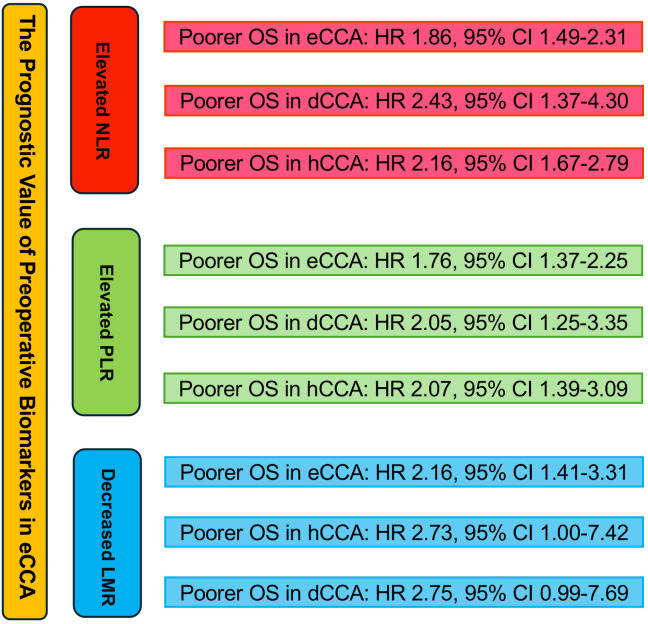
Graphical abstract summarizing the impact of NLR, PLR, and LMR on overall survival in eCCA patients.

In 5 studies, the correlation between LMR and eCCA prognosis was investigated. Among them, in four studies, a decreased LMR emerged as an independent predictor of compromised OS among patients with eCCA. Conversely, in 1 study, the impact of decreased LMR on OS did not reach statistical significance. The pooled analysis of all 5 studies revealed that LMR values below the defined cut-off values were indicative of poorer OS (HR 2.16, 95% CI 1.41–3.31, p < 0.001), albeit with moderate heterogeneity (**Figure 4**). The figure abstract for this study is presented in **Figure 5**.

### 3.6 Subgroup analyses of correlation between the NLR and OS of eCCA patients

The subgroup analysis for eCCA subtypes is as follows.

In 8 studies, eCCA was analyzed without further categorization. The results revealed that elevated NLR was identified as an independent predictor for impaired OS in patients with eCCA, with an HR of 1.55 (95% CI 1.32–1.82, p< 0.001), showing low heterogeneity (I2 = 14.7%). In 7 studies, the focus was on the distal CCA subtype within eCCA. The results indicated that elevated NLR was identified as an independent predictor for impaired OS in patients with eCCA, with an HR of 2.43 (95% CI 1.37–4.30, p= 0.002), exhibiting high heterogeneity. In 5 studies, the emphasis was on the hilar CCA subtype within eCCA. The findings demonstrated that elevated NLR was recognized as an independent predictor for impaired OS in patients with hilar CCA, with an HR of 2.16 (95% CI 1.67–2.79, p< 0.001), exhibiting low heterogeneity (I2 = 18.6%). The results of the subgroup analysis are presented in **Supplementary Figures 1**-**3**.

### 3.7 Subgroup analyses of correlation between the PLR and OS of eCCA patients

In 5 studies, eCCA was analyzed without further categorization. The findings indicated that elevated PLR was recognized as an independent predictor for impaired OS in patients with eCCA, with an HR of 1.41 (95% CI 1.09, 1.81, p < 0.001), displaying moderate heterogeneity(I2 = 44.6%). In 5 studies, the emphasis was on the distal CCA subtype within eCCA. The results indicated that elevated PLR was identified as an independent predictor for impaired OS in patients with eCCA, with an HR of 2.05 (95% CI 1.25, 3.35, p= 0.002), showing moderate heterogeneity(I2 = 45.9%). In 5 studies, the focus was on the hilar CCA subtype within eCCA. The findings demonstrated that elevated PLR was identified as an independent predictor for impaired OS in patients with hilar CCA, with an HR of 2.07 (95% CI 1.39, 3.09, p < 0.001), exhibiting moderate heterogeneity(I2 = 58.4%). The results of the subgroup analysis are presented in **Supplementary Figures 4**-**6**.

### 3.8 Subgroup analyses of correlation between the LMR and OS of eCCA patients

In studies investigating the relationship between LMR and OS in eCCA patients, 2 studies confined the scope of eCCA to hilar CCA. The conclusion from these two studies is that a decreased LMR leads to poorer OS, with an HR of 2.73 (95% CI 1.00, 7.42, P=0.049, I2 = 55%). In studies examining the correlation between LMR and OS in eCCA patients, two studies specifically focused on distal CCA. These studies concluded that a reduced LMR is associated with worse OS, with an HR of 2.75 (95% CI 0.99, 7.69 p=0.053, I2 = 62.6%). The results of the subgroup analysis are presented in **Supplementary Figures 7**, **8**.

### 3.9 Sensitivity analyses

In the sensitivity analyses, we employed a random-effects model, systematically excluding each study in turn, to ascertain the robustness of the prognostic role of NLR, PLR, and LMR in the overall survival (OS) of CCA. These sensitivity analyses were conducted using StataMP 17 software (StataCorp. 2022. Stata Statistical Software: Release 17.). The results reaffirmed the reliability of our findings. Moreover, detailed sensitivity analyses for each prognostic factor can be found in the supplementary materials (see **Supplementary Figures 9**-**11**).

### 3.10 Publication bias

In studies examining the association between NLR and OS, the asymmetrical distribution of funnel plots suggested a significant risk of publication bias. However, Egger’s regression test indicated that publication bias was significant, with p-values < 0.05(**Supplementary Figure 12**, **13**). To investigate the sources of publication bias, we divided all studies into three subgroups: eCCA, hCCA, and dCCA, and conducted Egger tests separately for each subgroup. For the first two subgroups, the Egger test’s p-values were greater than 0.05(eCCA: p=0.744, hCCA: p=0.824), indicating no significant publication bias (**Supplementary Figures 14**-**17**). However, for the dCCA subgroup, the Egger test’s p-value was less than 0.05, suggesting a significant presence of publication bias (**Supplementary Figures 18**, **19**).

In studies exploring the correlation between PLR and OS, the symmetrical distribution of funnel plots indicated no notable risk of publication bias. Furthermore, Egger’s regression test revealed an insignificant presence of publication bias, with p-values = 0.453 (**Supplementary Figures 20**, **21**).

In studies investigating the correlation between LMR and OS, the symmetrical distribution of funnel plots suggested no significant risk of publication bias. Additionally, Egger’s regression test indicated an insignificant presence of publication bias, with p-values = 0.053 (**Supplementary Figures 22**, **23**).

## 4 Discussion

The host’s inflammatory response within the tumor microenvironment is widely acknowledged for its pivotal role in cancer growth and progression, as well as its connection to systemic inflammation (17). Neutrophils, acting as significant sources of cytokines, are intricately involved in tumor progression (18). Likewise, platelets serve as potent sources of cytokines, capable of binding various growth factors such as vascular endothelial growth factor and fibroblast growth factor, both crucial in tumor angiogenesis, proliferation, and metastasis (19). Monocytes are recognized for their secretion of several pro-inflammatory cytokines, which have been shown to have adverse effects on cancer prognosis (20). On the other hand, lymphocytes, particularly tumor-infiltrating lymphocytes, play a critical role in the host’s anti-tumoral response. Therefore, these indices provided by hematologic components could offer valuable insights into the host-tumor interaction (21).

The prognostic association between NLR and eCCA may be achieved through the following mechanisms. Neutrophils play pivotal roles in carcinogenesis, generating reactive oxygen species, matrix metalloproteinase, and reactive nitrogen species, facilitating tumor initiation (22). They induce angiogenesis, compromise immunity, and impede CD8+ T cell function at metastatic sites (23). Conversely, lymphocytes induce tumor cell death, inhibit proliferation and migration, primarily mediated by CD8+ and CD4+ T cell interactions, releasing cytotoxic mediators and cytokines (24). Furthermore, mounting evidence suggests that an increased presence of tumor-infiltrating lymphocytes correlates with improved patient prognosis (25). Higher tumor-infiltrating lymphocytes correlate with better patient prognosis. According to the aforementioned mechanism, NLR is calculated by dividing the number of neutrophils by the number of lymphocytes. An increase in NLR can reflect either an enhanced neutrophil-dependent inflammatory response or a diminished lymphocyte-mediated antitumor immune response, both of which contribute to the poor prognosis of patients (15).

The mechanisms linking high PLR with poor prognosis have become increasingly understood. Interleukin-1 and Interleukin-6 stimulate megakaryocyte proliferation and differentiation into platelets (26). Furthermore, platelets are critical sources of growth factors, such as transforming growth factor β (TGF-β), vascular endothelial growth factor (VEGF), platelet-derived growth factor (PDGF), and platelet factor 4 (PF4) (27, 28). These factors promote angiogenesis and facilitate tumor progression and hematogenous metastasis. This association between elevated PLR and adverse prognosis has been observed across various cancers. An elevated PLR indicates the activation of transcription factors involved in the inflammatory response, such as signal transducer and activator of transcription 3 (STAT3), hypoxia-inducible factor 1a (HIF1a), and nuclear factor-kB (NF-kB) (29). These transcription factors lead to the secretion of pro-inflammatory cytokines, including TNF-α, IL-1β, and IL-6, which further promote tumor growth (30). An elevated PLR might, therefore, serve as a surrogate marker for the activity of transcription factors associated with cancer progression in CCA.

The link between decreased LMR in preoperative patients and poor prognosis is not fully understood, but it may be related to inflammation in the tumor microenvironment. A decreased LMR may reflect impaired immune function, characterized by lymphocytopenia and monocyte proliferation. Preoperative lymphocyte count has been shown to be a good indicator of tumor prognosis, reflecting the overall state of immune function and suggesting that lymphocyte-mediated cytotoxicity can inhibit cancer growth and metastasis (31). Thus, lymphopenia is considered a marker of host immunological incompetence. Conversely, the presence of monocytes, particularly tumor-associated macrophages (TAMs), contribute to cancer progression by promoting angiogenesis and lymphangiogenesis, leading to increased tumor cell proliferation, enhanced intravascular fluid flow, and higher rates of distant metastasis (32). A decreased LMR might, therefore, serve as a surrogate marker for the activity of transcription factors associated with cancer progression in CCA. The interplay between NLR, PLR, and LMR in eCCA reflects the balance between the inflammatory response and immune surveillance within the tumor microenvironment, significantly impacting tumor progression and patient prognosis. Elevated NLR and PLR, along with decreased LMR, collectively indicate an enhanced inflammatory response and impaired immune surveillance, both of which play critical roles in promoting tumor growth and metastasis. These ratios provide valuable insights into the systemic inflammatory and immune status, aiding in the prognostic assessment and treatment decision-making for eCCA patients.

While lymph node metastasis, poor histological differentiation, positive surgical margins, and pre-/post-CA19–9 levels are known prognostic indicators in ECC patients, their utility in guiding preoperative treatment strategies is limited as they are mostly obtained postoperatively. Consequently, there is a pressing need for effective preoperative prognostic markers to inform treatment decisions. In prior research, NLR, PLR, and LMR have emerged as noteworthy prognostic markers in patients with various digestive system tumors (33–35). Nonetheless, conflicting findings exist regarding the prognostic implications of these preoperative systemic inflammatory parameters in CCA (36, 37). Our results show that among the 20 studies included in the analysis, NLR, PLR, and LMR are all associated with OS and can potentially be used as prognostic indices in eCCA. Our study is the first meta- analysis to investigate the prognostic value of preoperative biomarkers NLR, PLR, and LMR specifically in extrahepatic cholangiocarcinoma. The clinical significance of our study lies in the fact that NLR, PLR, and LMR are all simple, inexpensive, and reproducible measurements that can be performed preoperatively. Their results can provide relatively accurate predictive indications of patient prognosis, enabling us to devise better strategies for patient management. In the pathophysiological context of eCCA, NLR, PLR, and LMR serve as integrated biomarkers by encapsulating the intricate balance between pro-inflammatory and anti-tumor immune responses. Elevated NLR and PLR, coupled with decreased LMR, signify heightened systemic inflammation and weakened immune defense mechanisms, which are pivotal in tumor progression and metastasis (38). These biomarkers not only mirror the underlying inflammatory state but also correlate with disease stage and prognosis, offering valuable insights into the overall tumor biology and aiding clinicians in predicting patient outcomes and tailoring personalized treatment strategies.

The limited adoption of biomarkers such as NLR, PLR, and LMR in clinical practice, despite their established prognostic value, is due to several factors. Variability in cut-off values, differences in measurement protocols, and the impact of treatment regimens can affect these biomarkers’ reliability and consistency. Additionally, the lack of standardized guidelines and insufficient multicentric validation further impede their integration into routine clinical decision-making. Addressing these issues requires standardized measurement practices, comprehensive validation studies, and a better understanding of how these biomarkers can be effectively utilized alongside other clinical variables (39). Preoperative measurements of NLR, PLR, and LMR can be significantly influenced by treatments like chemotherapy and radiotherapy, as well as by the presence of conditions such as cholangitis or bile duct stents. These factors can impact the biomarkers’ levels and their interpretation (40). Therefore, it is crucial to standardize the timing of blood sample collection relative to treatments and to consider these variables when assessing the biomarkers’ prognostic value. Addressing these issues can enhance the accuracy and clinical utility of NLR, PLR, and LMR in predicting outcomes in eCCA. Furthermore, it is essential to explore how NLR, PLR, and LMR change throughout diagnosis, treatment, surgery, and disease progression. Biomarkers like NLR, PLR, and LMR can fluctuate due to disease progression, treatment interventions, and clinical conditions. Single-timepoint measurements may not fully capture these variations. Multiple measurements taken at different stages—such as preoperative, during treatment, and post-surgery—can provide a more comprehensive understanding of their prognostic value. Evaluating these biomarkers at various timepoints may enhance the accuracy of prognostic predictions and lead to more personalized treatment strategies. Future research should focus on integrating longitudinal data to improve the clinical utility of these biomarkers and address the limitations associated with single-timepoint assessments.

In eCCA, the interplay between NLR, PLR, and LMR is crucial in understanding tumor progression. Elevated NLR and PLR, along with decreased LMR, highlight the enhanced inflammatory response and impaired immune surveillance specific to eCCA. Neutrophils and platelets are involved in eCCA growth and metastasis, while lymphocytes play a protective role. These biomarkers—NLR, PLR, and LMR—reflect the systemic inflammatory and immune status in eCCA, providing valuable insights for prognostic assessment and tailored treatment strategies in eCCA patients. The prognostic implications of NLR, PLR, and LMR in eCCA show both similarities and differences when compared to other types of cancers. For instance, in hepatocellular carcinoma (HCC), elevated NLR has been linked to poor overall survival and increased recurrence rates (41), like findings in eCCA. In colorectal cancer, high PLR is associated with worse prognosis, reflecting systemic inflammation and tumor progression, much like in eCCA (42). Conversely, in lung cancer, a low LMR indicates a poor prognosis due to a compromised immune response, akin to its role in eCCA (43).

Like all meta-analyses based on a limited pool of literature, our study has several limitations. Firstly, the retrospective nature of all included studies may introduce selection bias in the published data. Secondly, the variability in cutoff values for NLR, PLR, and LMR, as ratio indicators, across the included original studies underscores the need for more large-scale prospective research to establish optimal cutoff values for these indicators. Additionally, certain outcomes, such as the correlation between the PLR and OS of eCCA patients, exhibited high heterogeneity due to differences in study populations, measurement methods, disease stages, treatment regimens, and reporting practices. Finally, the predominance of studies from Asian populations may limit the generalizability of our findings.

## 5 Conclusions

In conclusion, our study underscores the clinical significance of preoperative evaluation of NLR, PLR, and LMR in predicting patient prognosis. Elevated NLR values exceeding defined thresholds were associated with poorer overall survival (OS), mirroring the association between poorer OS and PLR values surpassing defined cut-off levels, as well as LMR values falling below defined thresholds. Large-scale prospective cohort studies are essential to validate the independent prognostic significance of NLR, PLR, and LMR in eCCA.

## Data Availability

The original contributions presented in the study are included in the article/**Supplementary Material**. Further inquiries can be directed to the corresponding authors.

## Supplementary material

Supplementary Figure 1 NLR and Overall Survival (OS) in eCCA Patients.

Supplementary Figure 2 NLR and OS in Distal CCA Subtype within eCCA.

Supplementary Figure 3 NLR and OS in Hilar CCA Subtype within eCCA.

Supplementary Figure 4 PLR and OS in eCCA Patients.

Supplementary Figure 5 PLR and OS in Distal CCA Subtype within eCCA.

Supplementary Figure 6 PLR and OS in Hilar CCA Subtype within eCCA.

Supplementary Figure 7 LMR and OS in Hilar CCA Subtype within eCCA.

Supplementary Figure 8 LMR and OS in Distal CCA Subtype within eCCA.

Supplementary Figure 9 Sensitivity Analysis of NLR in OS of eCCA Patients.

Supplementary Figure 10 Sensitivity Analysis of PLR in OS of eCCA Patients.

Supplementary Figure 11 Sensitivity Analysis of LMR in OS of eCCA Patients.

Supplementary Figure 12 Publication Bias in NLR and OS Studies.

Supplementary Figure 13 Publication Bias in NLR and OS Studies.

Supplementary Figure 14 Publication Bias in NLR and OS Subgroup Studies.

Supplementary Figure 15 Publication Bias in NLR and OS Subgroup Studies.

Supplementary Figure 16 Publication Bias in NLR and OS Subgroup Studies.

Supplementary Figure 17 Publication Bias in NLR and OS Subgroup Studies.

Supplementary Figure 18 Publication Bias in NLR and OS Subgroup Studies.

Supplementary Figure 19 Publication Bias in NLR and OS Subgroup Studies.

Supplementary Figure 20 Publication Bias in PLR and OS Studies.

Supplementary Figure 21 Publication Bias in PLR and OS Studies.

Supplementary Figure 22 Publication Bias in LMR and OS Studies.

Supplementary Figure 23 Publication Bias in LMR and OS Studies.
