# Supplementary figures and images for: The prognostic value of preoperative peripheral blood inflammatory biomarkers in extrahepatic cholangiocarcinoma: a systematic review and meta-analysis
Source: Front Oncol. 2024 Aug 29;14:1437978. doi: 10.3389/fonc.2024.1437978 (PMC11390462; doi:10.3389/fonc.2024.1437978)

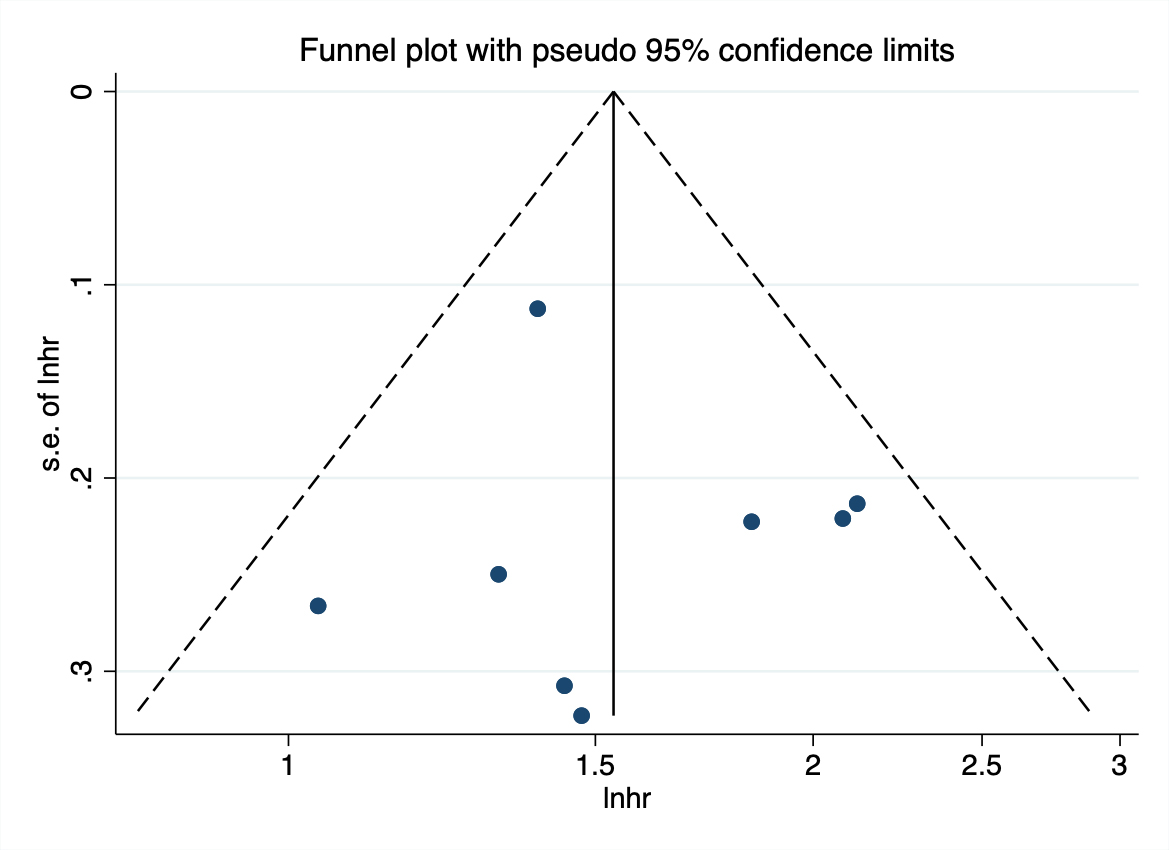

Supplement: Supplementary Figure 1 — NLR and Overall Survival (OS) in eCCA Patients. [file DataSheet1.zip › Supplementary file/Supplementary Figures 15.jpg]

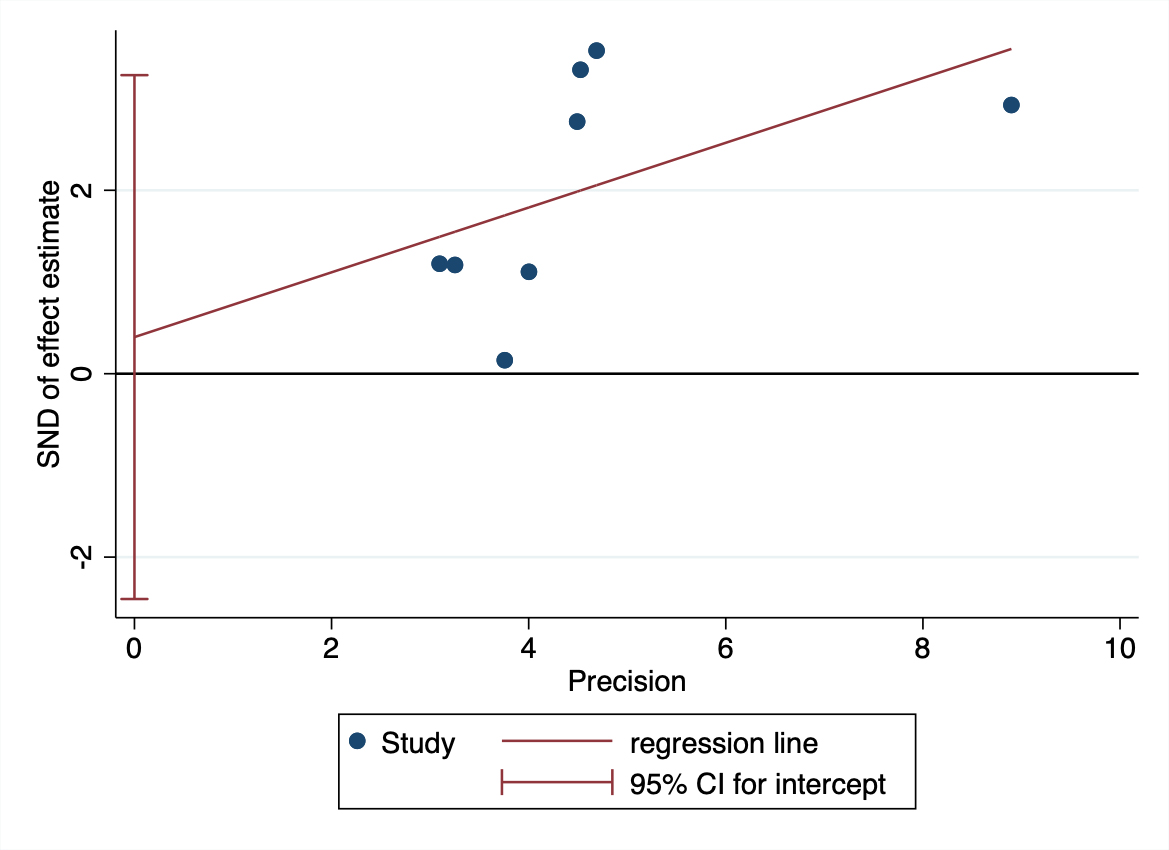

Supplement: Supplementary Figure 1 — NLR and Overall Survival (OS) in eCCA Patients. [file DataSheet1.zip › Supplementary file/Supplementary Figures 14.jpg]

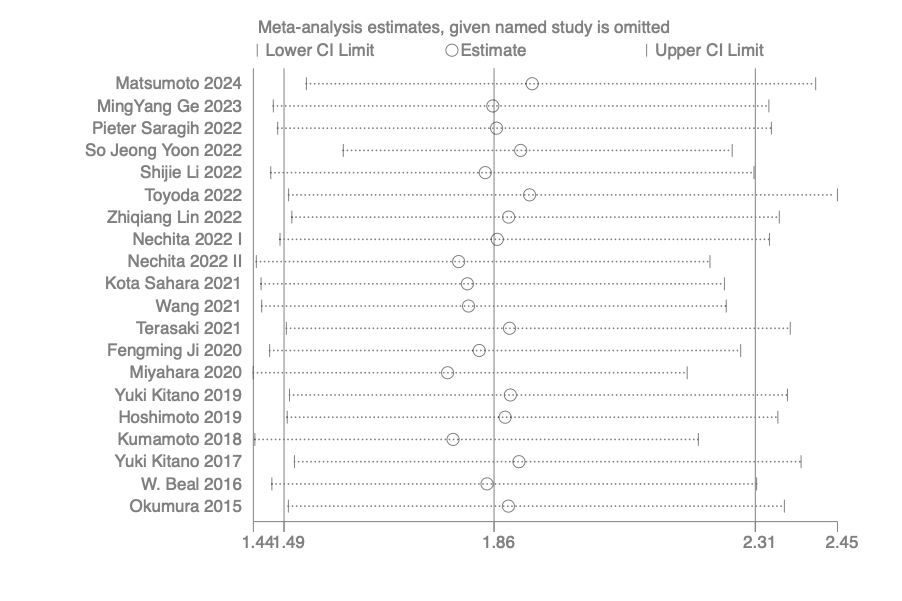

Supplement: Supplementary Figure 1 — NLR and Overall Survival (OS) in eCCA Patients. [file DataSheet1.zip › Supplementary file/Supplementary Figures 9.jpg]

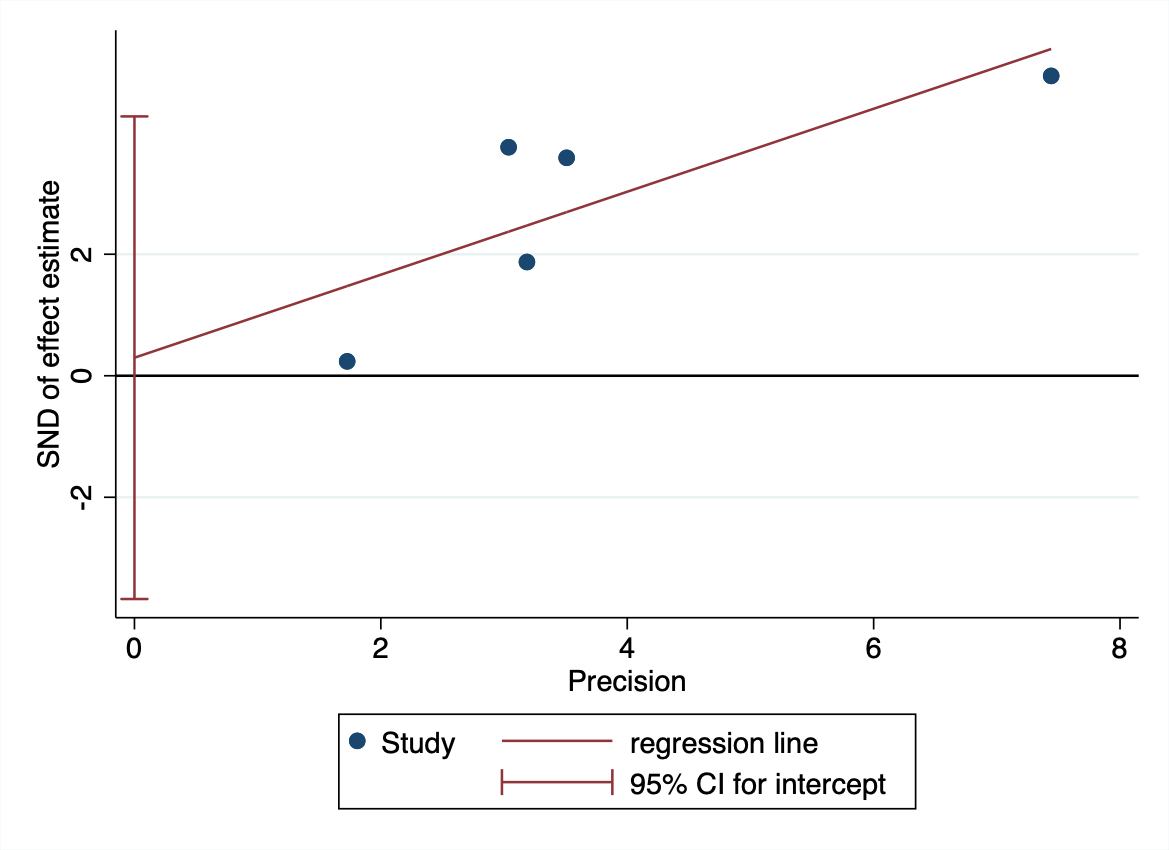

Supplement: Supplementary Figure 1 — NLR and Overall Survival (OS) in eCCA Patients. [file DataSheet1.zip › Supplementary file/Supplementary Figures 16.jpg]

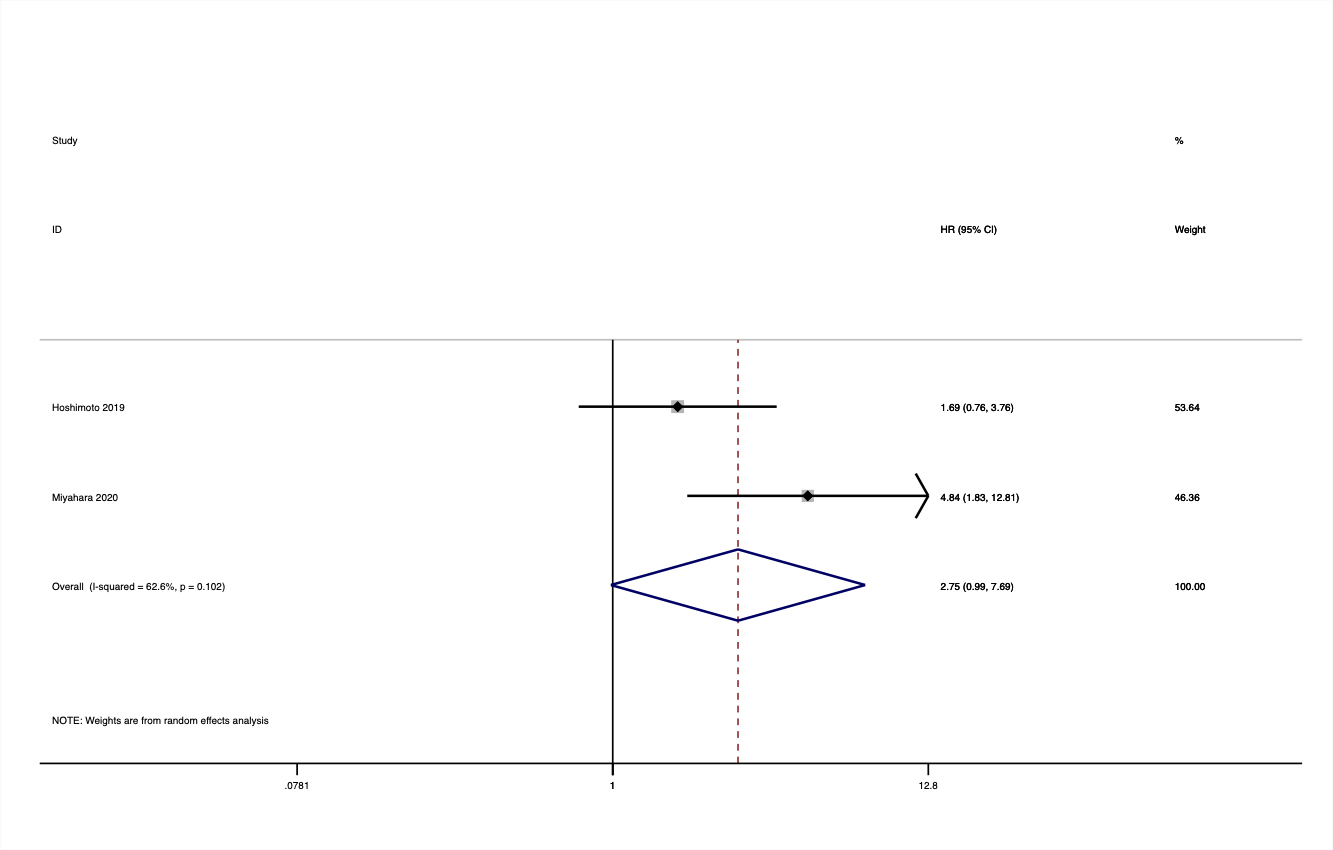

Supplement: Supplementary Figure 1 — NLR and Overall Survival (OS) in eCCA Patients. [file DataSheet1.zip › Supplementary file/Supplementary figure8.jpg]

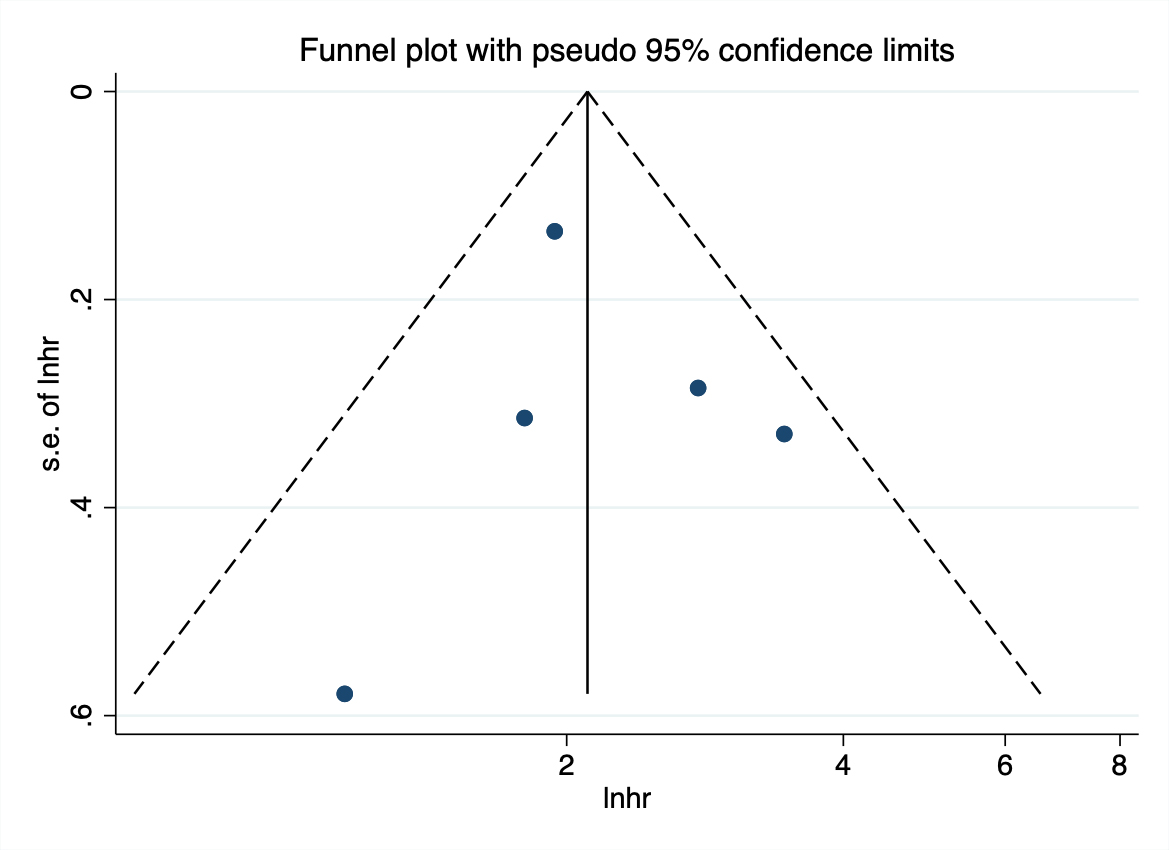

Supplement: Supplementary Figure 1 — NLR and Overall Survival (OS) in eCCA Patients. [file DataSheet1.zip › Supplementary file/Supplementary Figures 17.jpg]

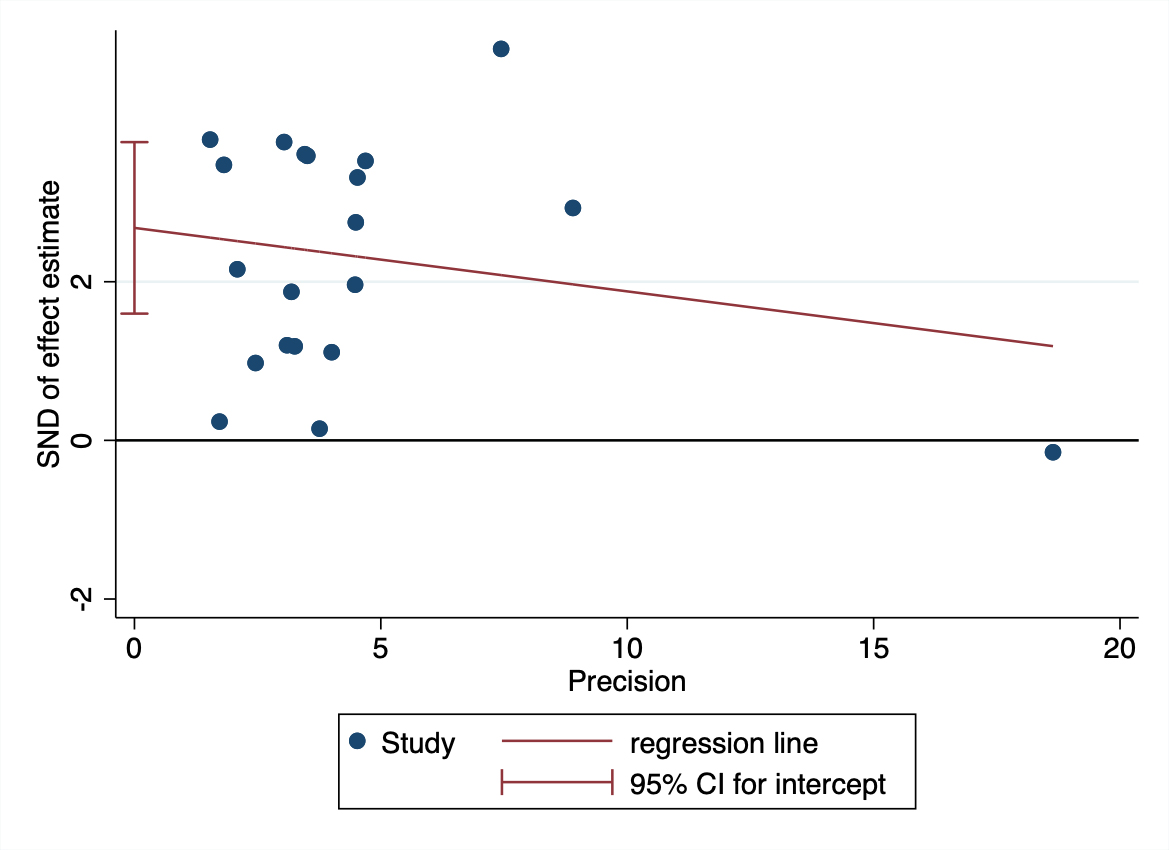

Supplement: Supplementary Figure 1 — NLR and Overall Survival (OS) in eCCA Patients. [file DataSheet1.zip › Supplementary file/Supplementary Figures 13.jpg]

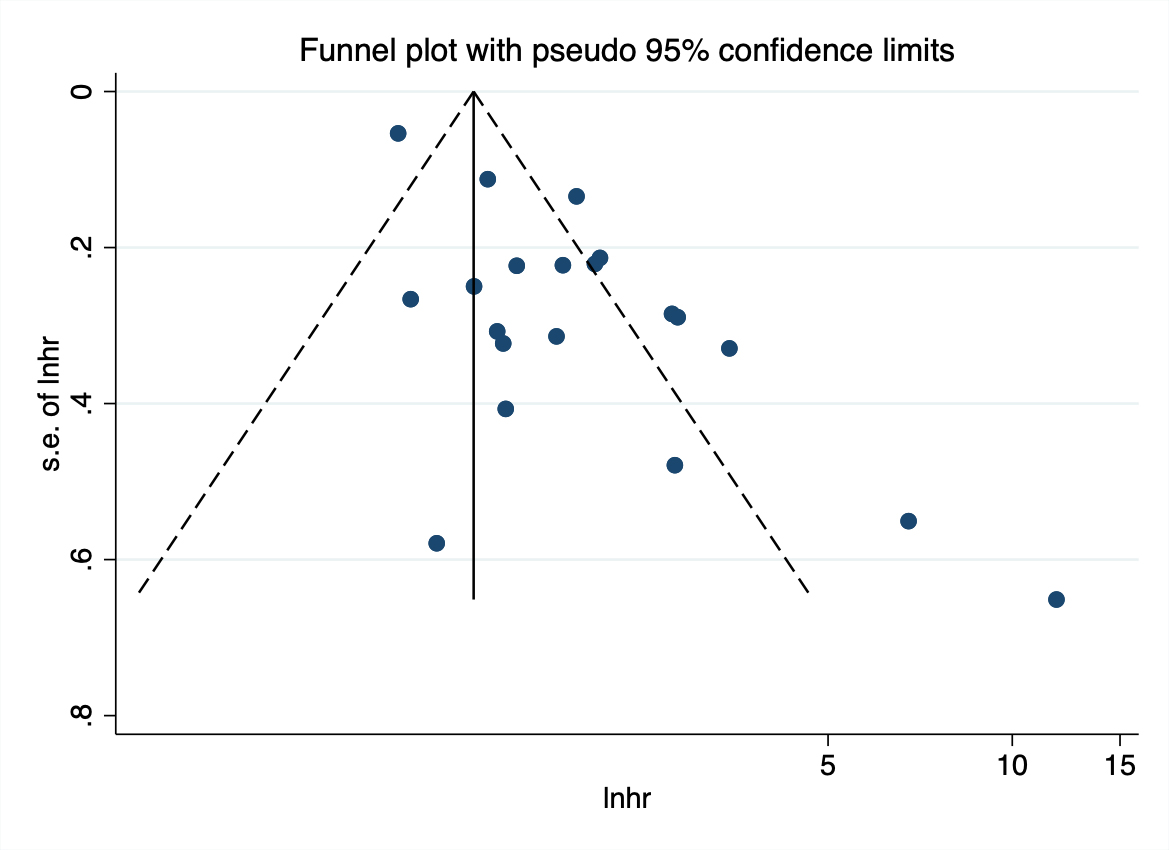

Supplement: Supplementary Figure 1 — NLR and Overall Survival (OS) in eCCA Patients. [file DataSheet1.zip › Supplementary file/Supplementary Figures 12.jpg]

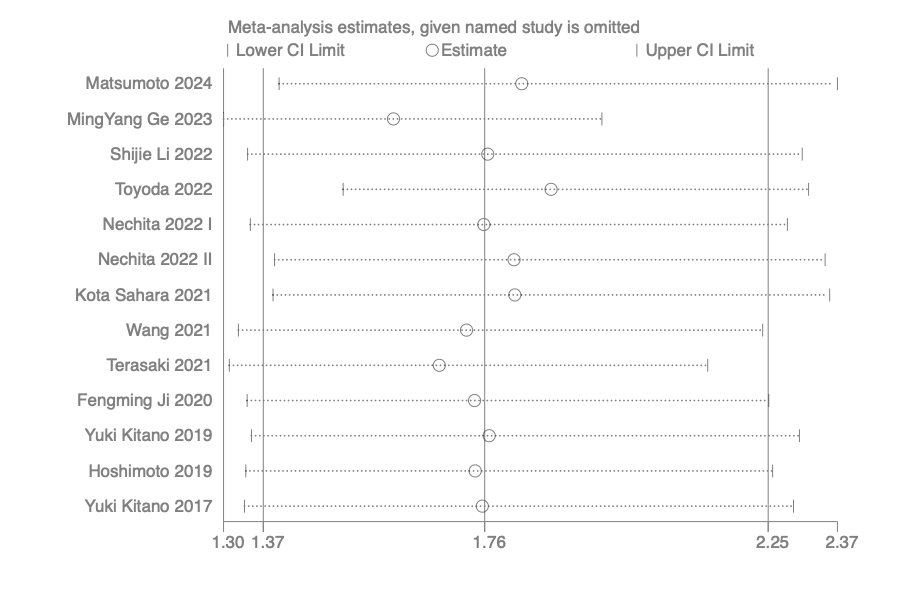

Supplement: Supplementary Figure 1 — NLR and Overall Survival (OS) in eCCA Patients. [file DataSheet1.zip › Supplementary file/Supplementary Figures 10.jpg]

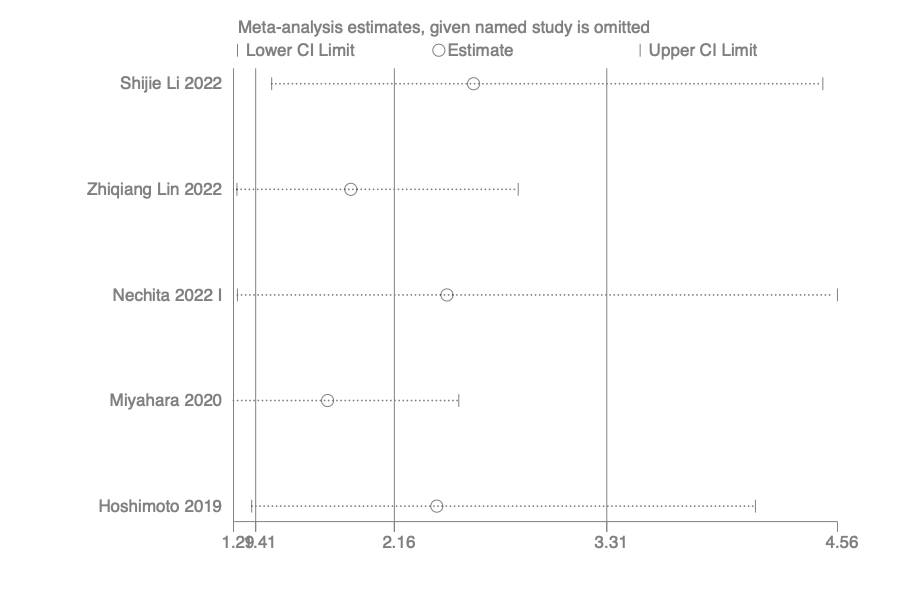

Supplement: Supplementary Figure 1 — NLR and Overall Survival (OS) in eCCA Patients. [file DataSheet1.zip › Supplementary file/Supplementary Figures 11.jpg]

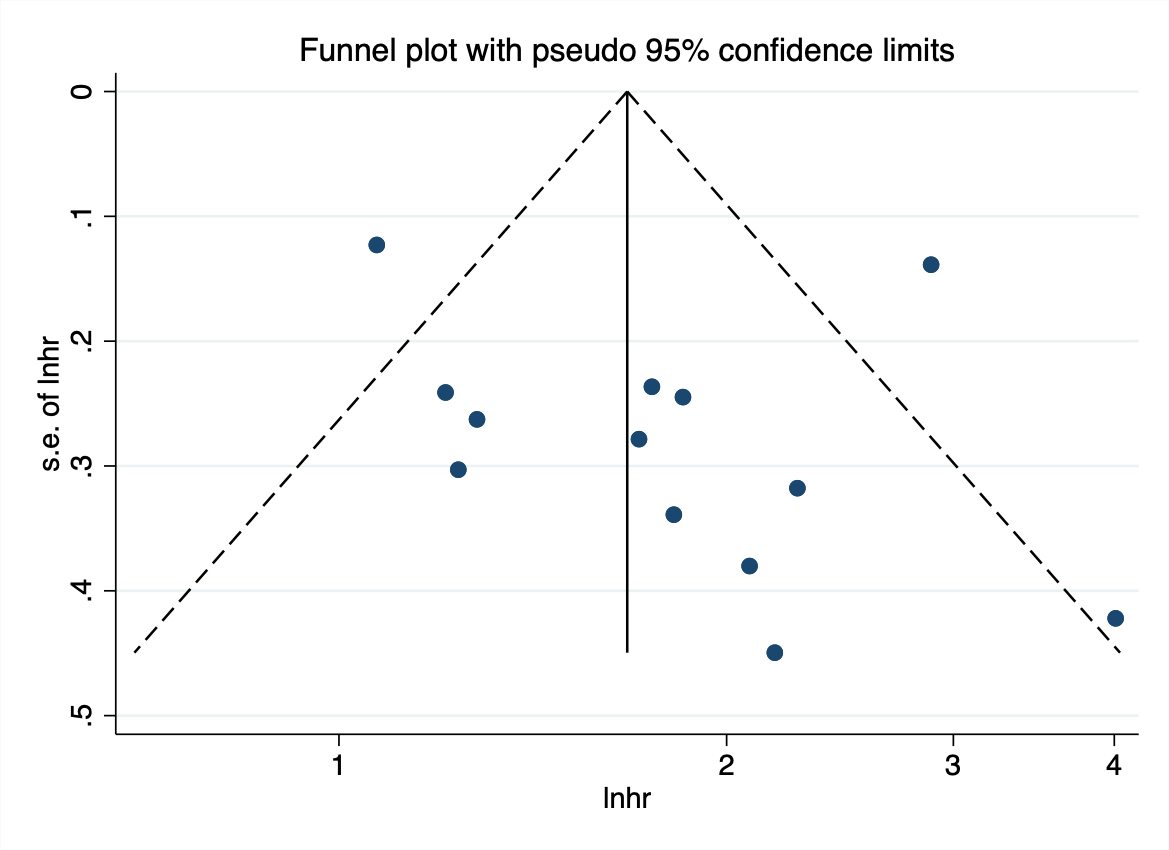

Supplement: Supplementary Figure 1 — NLR and Overall Survival (OS) in eCCA Patients. [file DataSheet1.zip › Supplementary file/Supplementary Figures 20.jpg]

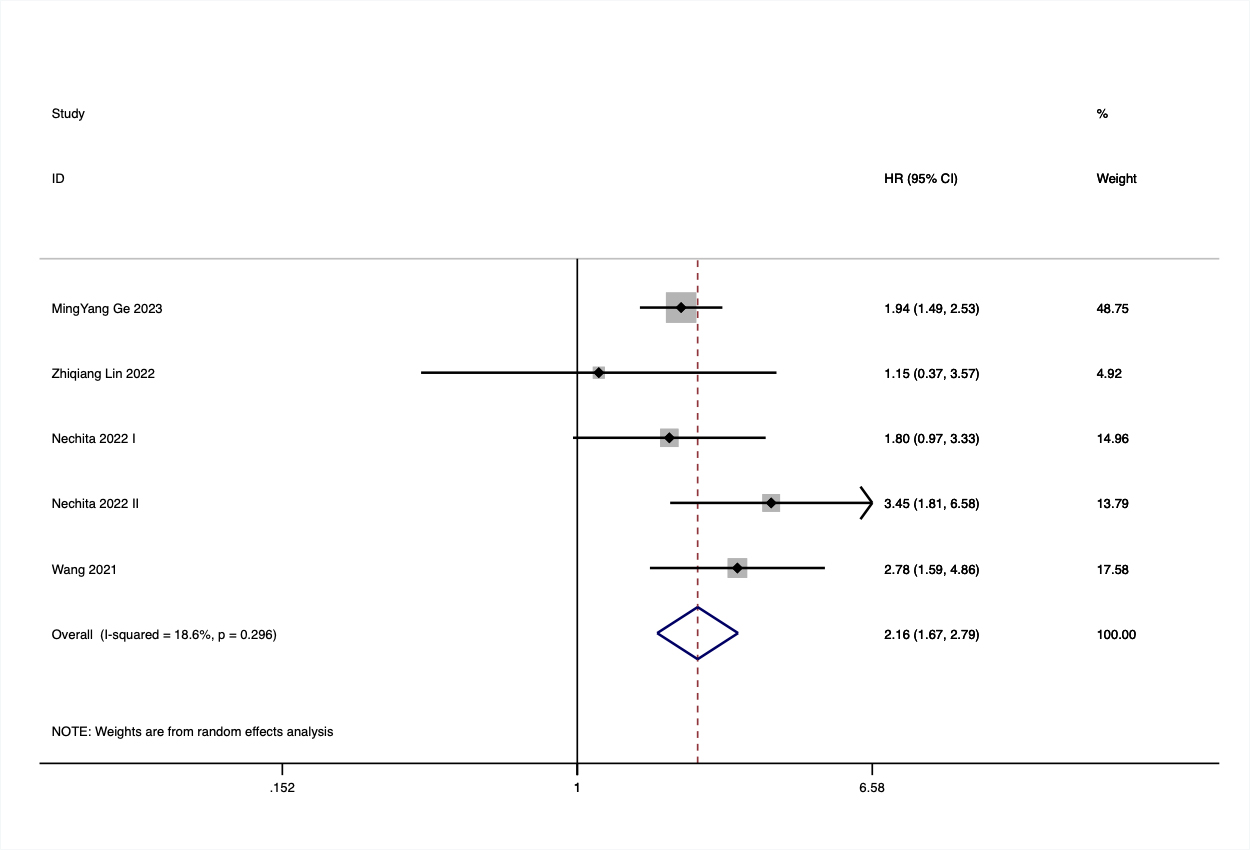

Supplement: Supplementary Figure 1 — NLR and Overall Survival (OS) in eCCA Patients. [file DataSheet1.zip › Supplementary file/Supplementary figure3.jpg]

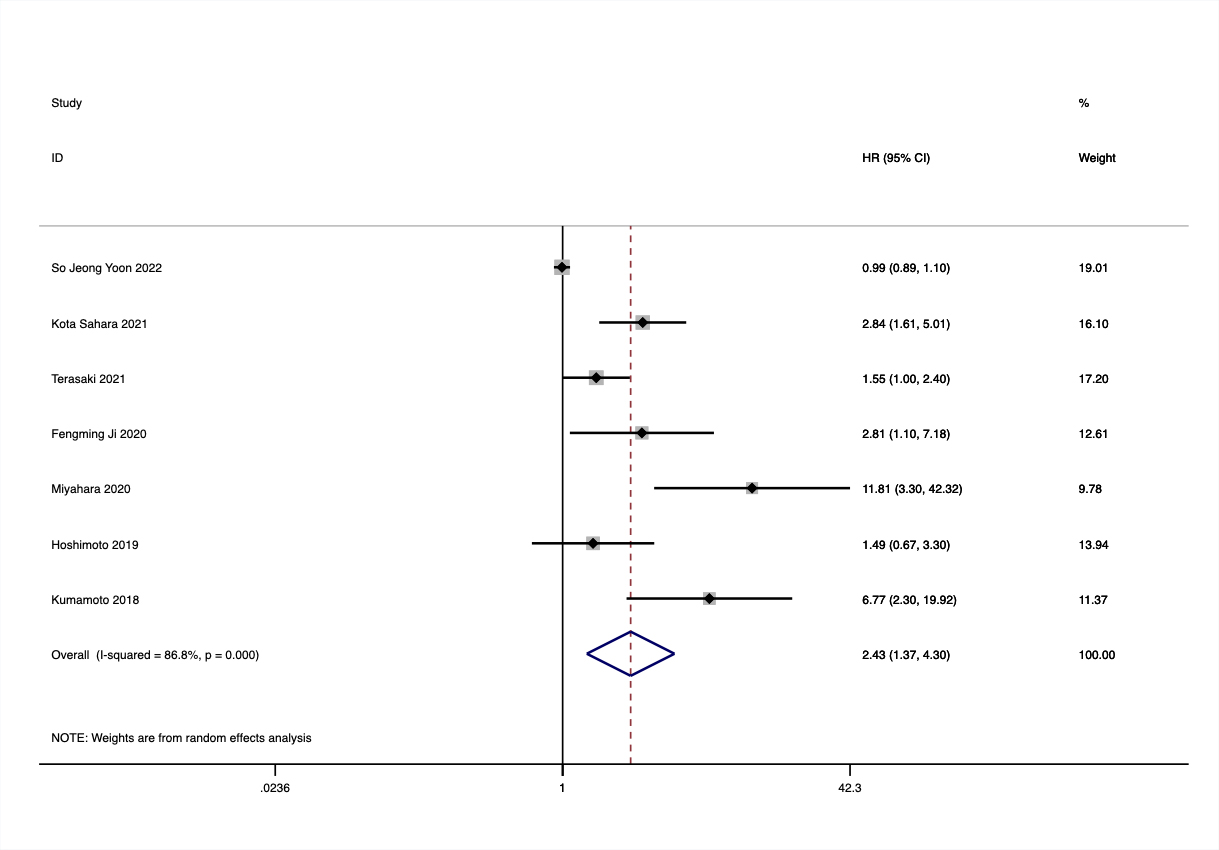

Supplement: Supplementary Figure 1 — NLR and Overall Survival (OS) in eCCA Patients. [file DataSheet1.zip › Supplementary file/Supplementary figure2.jpg]

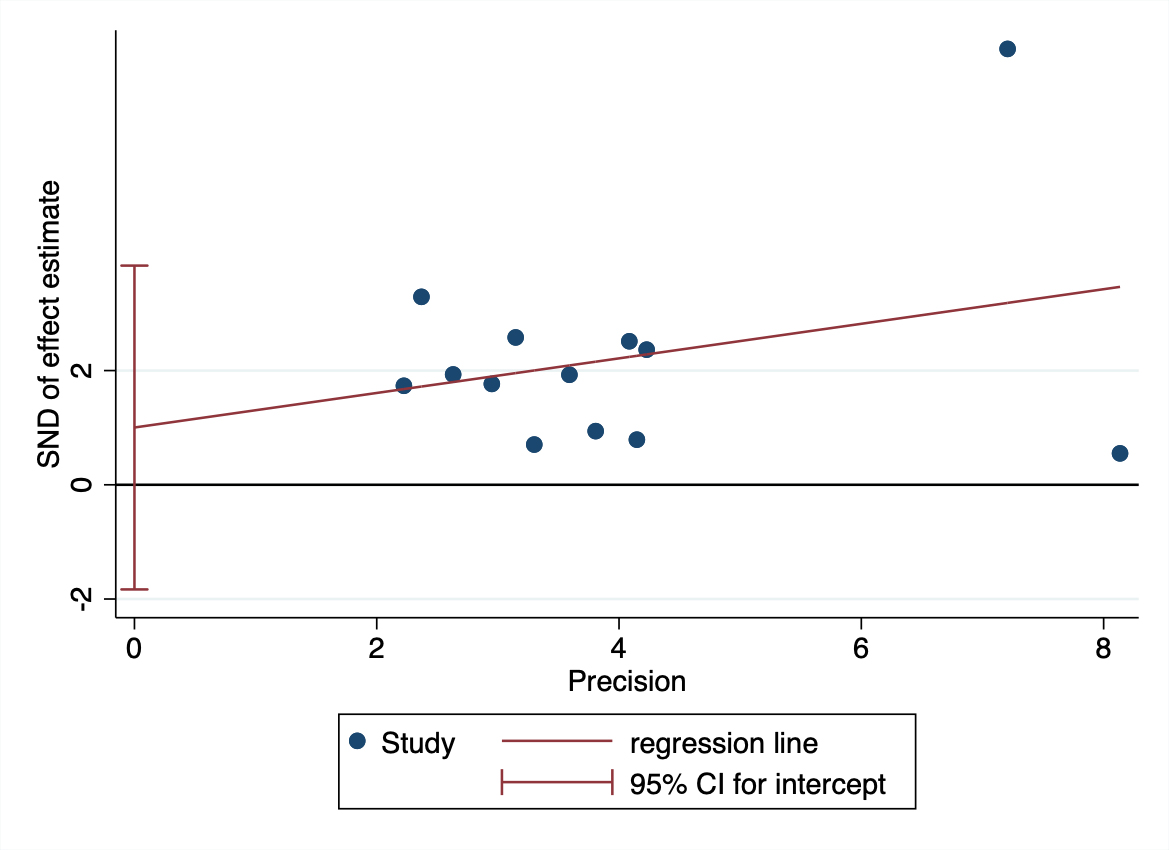

Supplement: Supplementary Figure 1 — NLR and Overall Survival (OS) in eCCA Patients. [file DataSheet1.zip › Supplementary file/Supplementary Figures 21.jpg]

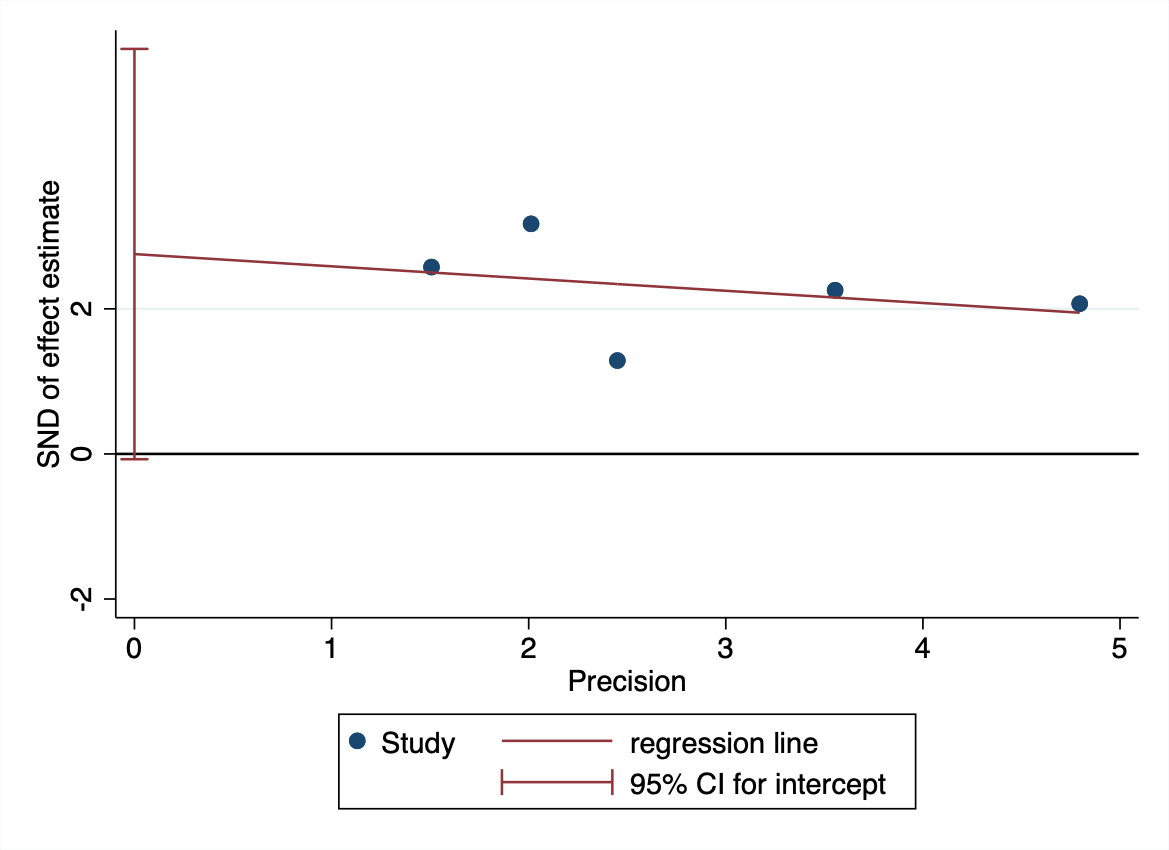

Supplement: Supplementary Figure 1 — NLR and Overall Survival (OS) in eCCA Patients. [file DataSheet1.zip › Supplementary file/Supplementary Figures 23.jpg]

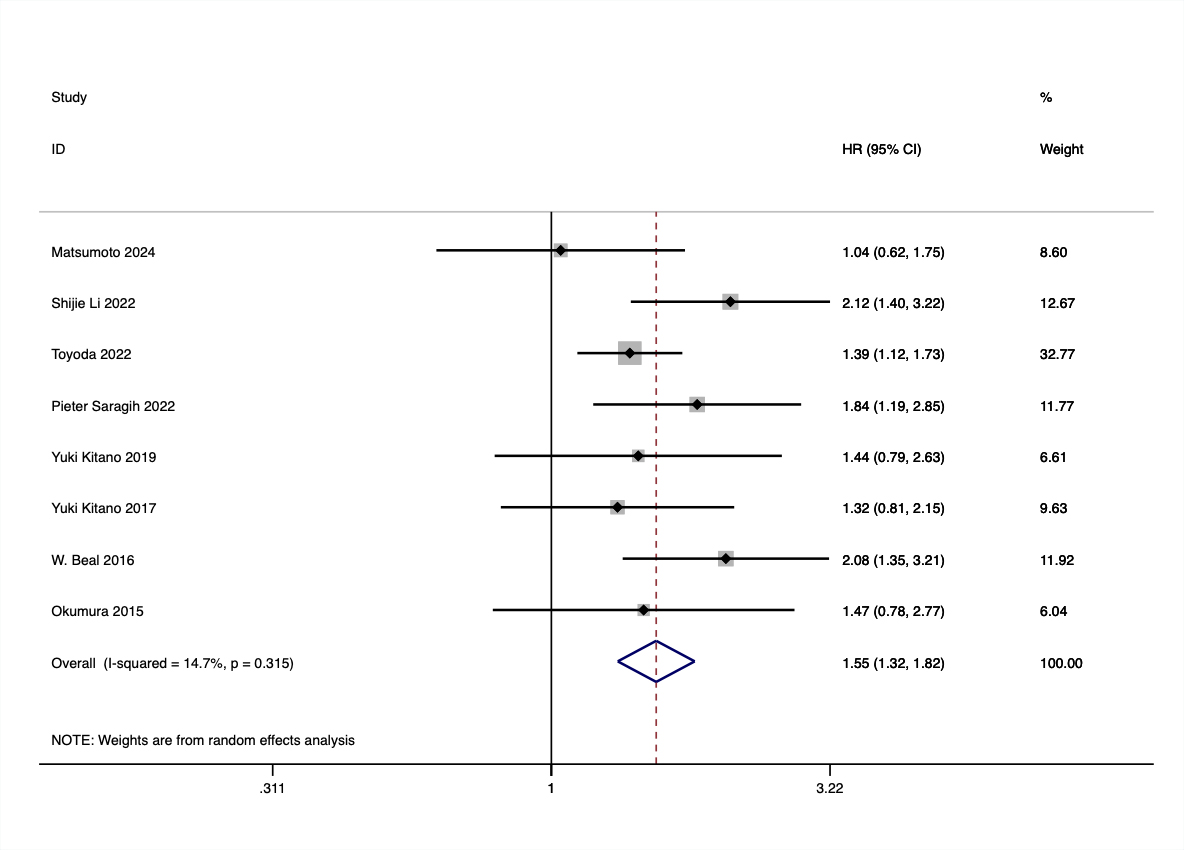

Supplement: Supplementary Figure 1 — NLR and Overall Survival (OS) in eCCA Patients. [file DataSheet1.zip › Supplementary file/Supplementary figure1.jpg]

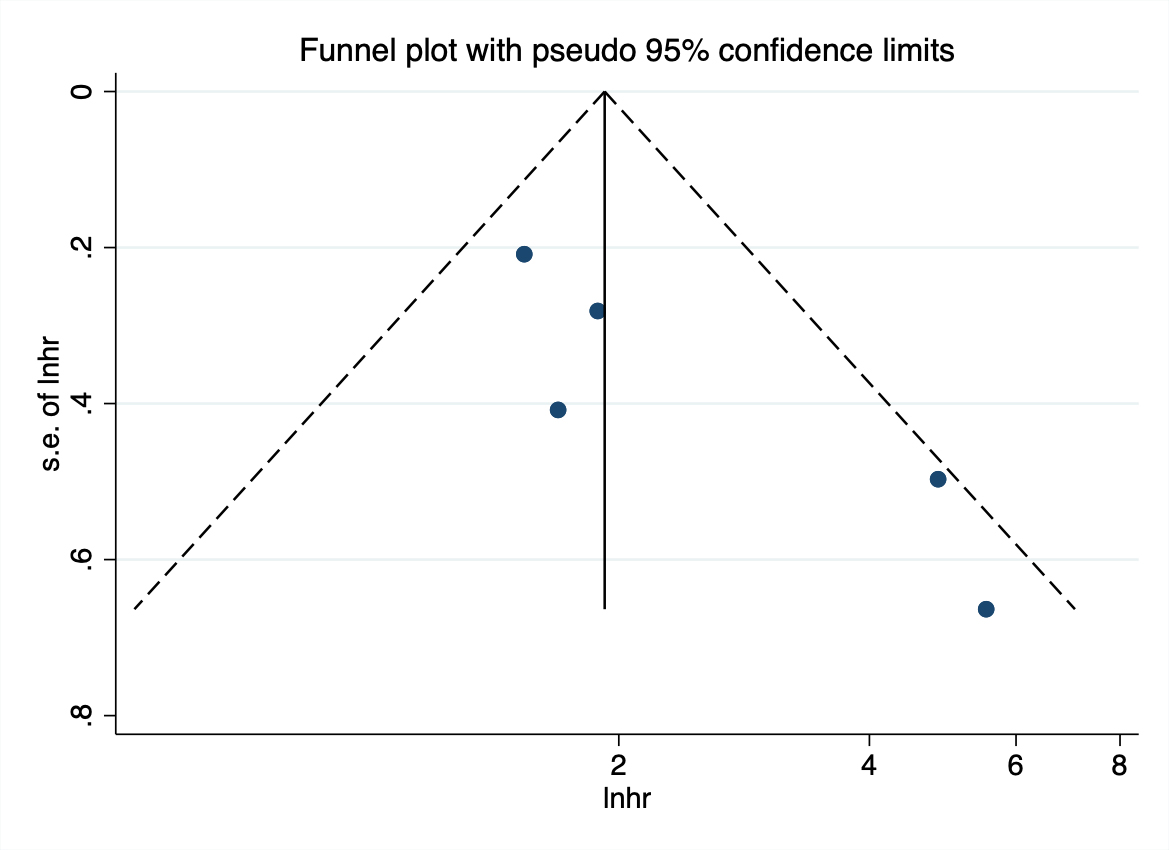

Supplement: Supplementary Figure 1 — NLR and Overall Survival (OS) in eCCA Patients. [file DataSheet1.zip › Supplementary file/Supplementary Figures 22.jpg]

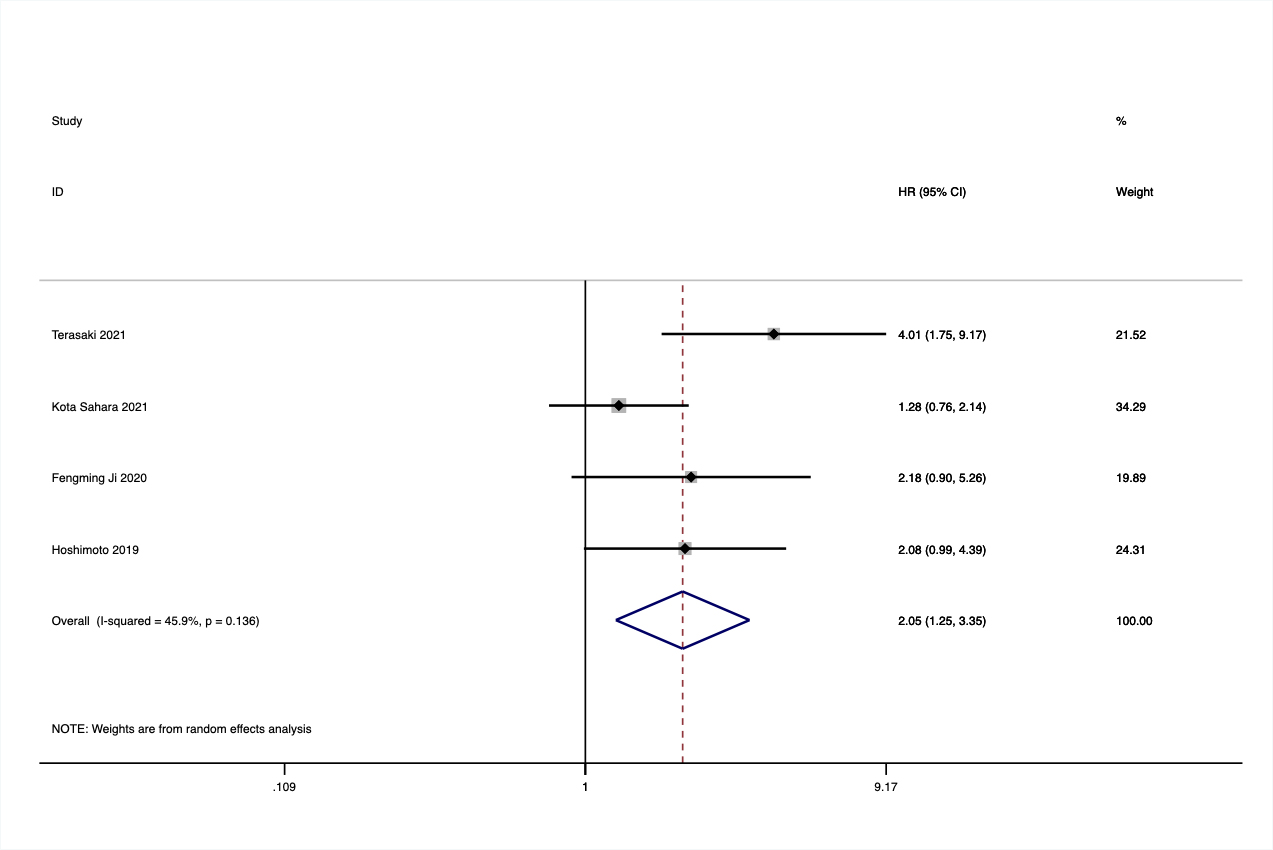

Supplement: Supplementary Figure 1 — NLR and Overall Survival (OS) in eCCA Patients. [file DataSheet1.zip › Supplementary file/Supplementary figure5.jpg]

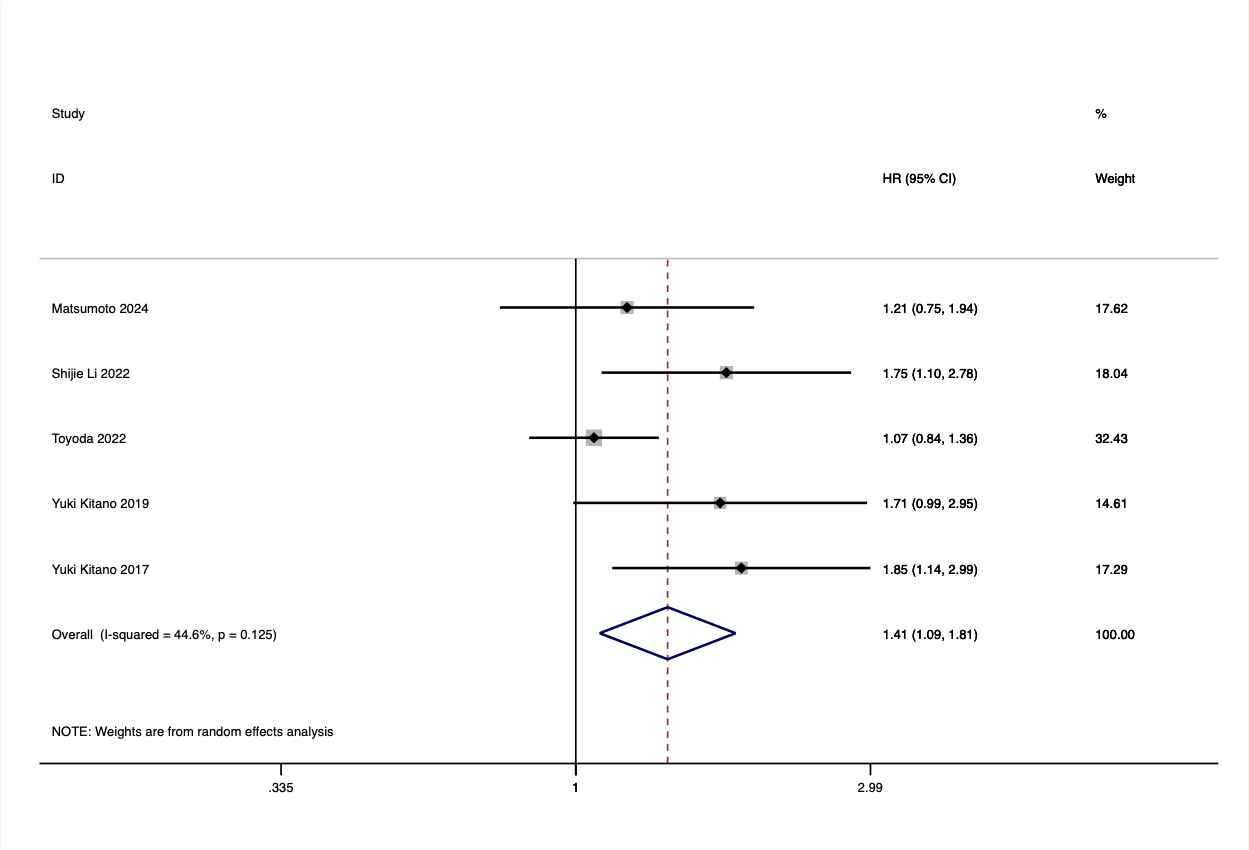

Supplement: Supplementary Figure 1 — NLR and Overall Survival (OS) in eCCA Patients. [file DataSheet1.zip › Supplementary file/Supplementary figure4.jpg]

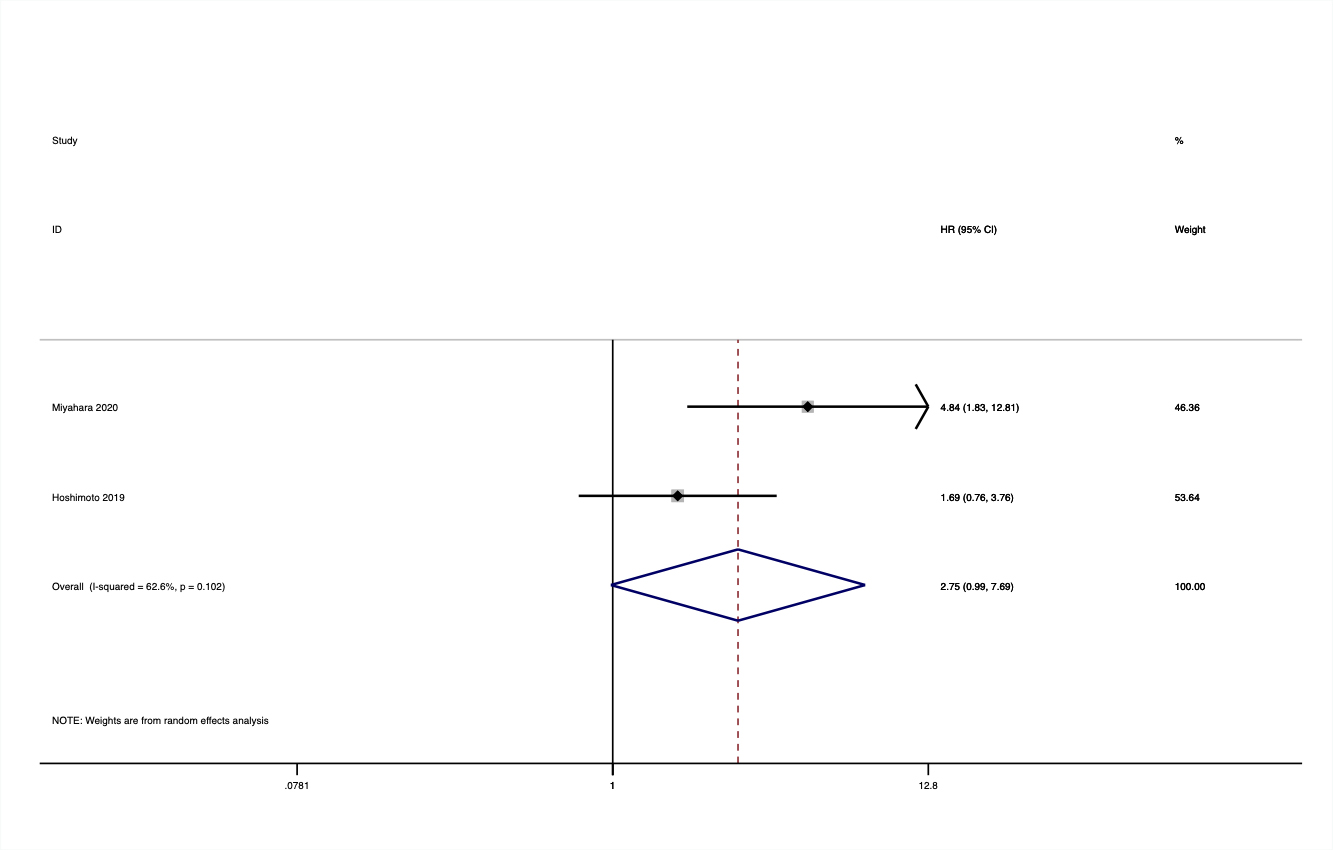

Supplement: Supplementary Figure 1 — NLR and Overall Survival (OS) in eCCA Patients. [file DataSheet1.zip › Supplementary file/Supplementary Figures 19.jpg]

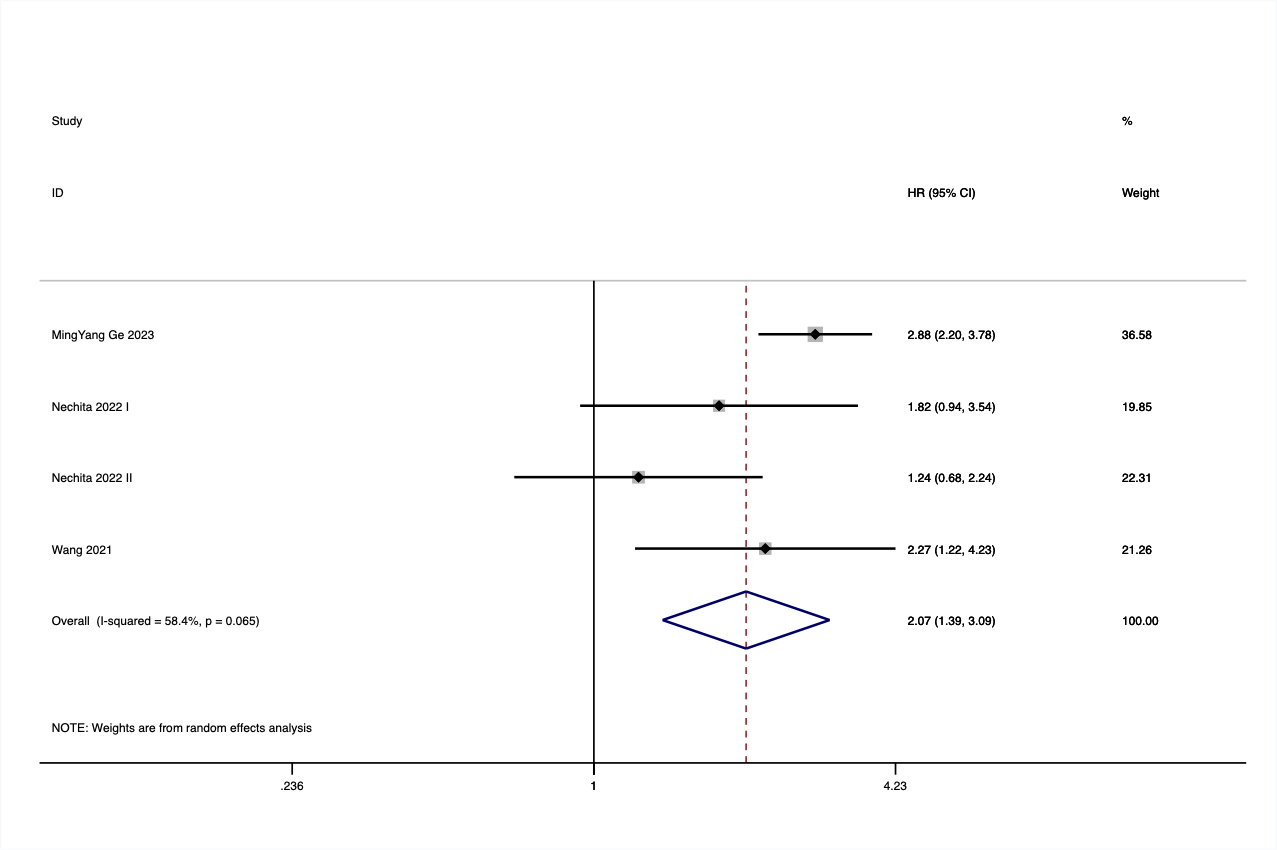

Supplement: Supplementary Figure 1 — NLR and Overall Survival (OS) in eCCA Patients. [file DataSheet1.zip › Supplementary file/Supplementary figure6.jpg]

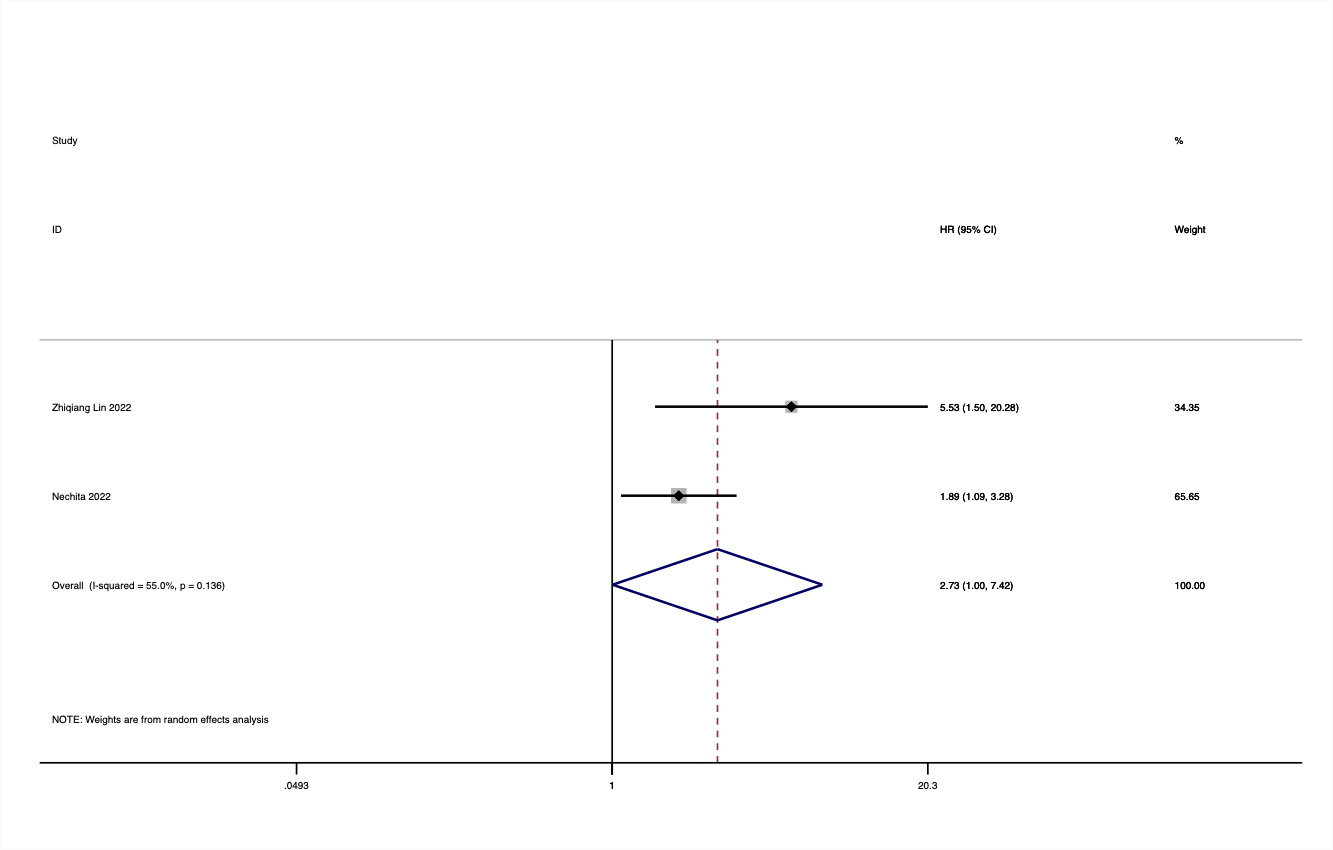

Supplement: Supplementary Figure 1 — NLR and Overall Survival (OS) in eCCA Patients. [file DataSheet1.zip › Supplementary file/Supplementary figure7.jpg]
